# Mitochondrial Metabolism in Astrocytes Regulates Brain Bioenergetics, Neurotransmission and Redox Balance

**DOI:** 10.3389/fnins.2020.536682

**Published:** 2020-11-05

**Authors:** Jordan Rose, Christian Brian, Aglaia Pappa, Mihalis I. Panayiotidis, Rodrigo Franco

**Affiliations:** ^1^Redox Biology Center, University of Nebraska-Lincoln, Lincoln, NE, United States; ^2^School of Veterinary Medicine and Biomedical Sciences, University of Nebraska-Lincoln, Lincoln, NE, United States; ^3^Department of Molecular Biology and Genetics, Democritus University of Thrace, Alexandroupolis, Greece; ^4^Department of Electron Microscopy & Molecular Pathology, Cyprus Institute of Neurology & Genetics, Nicosia, Cyprus

**Keywords:** glycolysis, astrocytes, bioenergetics, redox, fatty acid oxidation, cataplerotic, anaplerotic, mitochondria

## Abstract

In the brain, mitochondrial metabolism has been largely associated with energy production, and its dysfunction is linked to neuronal cell loss. However, the functional role of mitochondria in glial cells has been poorly studied. Recent reports have demonstrated unequivocally that astrocytes do not require mitochondria to meet their bioenergetics demands. Then, the question remaining is, what is the functional role of mitochondria in astrocytes? In this work, we review current evidence demonstrating that mitochondrial central carbon metabolism in astrocytes regulates overall brain bioenergetics, neurotransmitter homeostasis and redox balance. Emphasis is placed in detailing carbon source utilization (glucose and fatty acids), anaplerotic inputs and cataplerotic outputs, as well as carbon shuttles to neurons, which highlight the metabolic specialization of astrocytic mitochondria and its relevance to brain function.

## Introduction

The central nervous system (CNS) is a complex, integrated structure composed of diverse cell types. Neurons are highly specialized cells in charge of transmitting, processing, and storing information and thus have been considered the functional units of the CNS. However, research within the last few decades has demonstrated the critical roles non-neuronal cells play in CNS function. The non-neuronal cells of the CNS include macroglia (astrocytes, ependymal cells and oligodendrocytes) and microglia, which support essential functions such as the maintenance of neurotransmitter communication, metabolism, trophic support, formation of myelin sheath, wound healing, and immune surveillance (von Bernhardi et al., 2016).

Astrocytes originate from the neuroectoderm. Recent estimates suggest they account for between 19 and 40% of the glial population (with an estimated glial to neuron ratio of ∼1:1), and a strong regional variation within the CNS (von Bartheld et al., 2016). Astrocytes are a heterogenous population of cells with a diverse array of morphological, functional and molecular properties (Garcia-Marin et al., 2007). While the cellular identity of astrocytes is determined by their gene expression profile post-differentiation (intrinsic patterning) (Song et al., 2002; Krencik et al., 2011), the surrounding environment and neuronal circuitry further shapes, modifies, and maintains the form and functions of differentiated astrocytes (extrinsic factors) (Koulakoff et al., 2008; Yang et al., 2009; Farmer et al., 2016; Ben Haim and Rowitch, 2017). Structurally, astrocytes contain multiple radial processes that create diverse interfaces with other glia, neurons, and capillary endothelial cells. Astrocytes’ fine processes and endfeet envelope neuronal cell bodies, synapses, and blood vessels. In addition, an intracellular interface with other astrocytes or oligodendrocytes is generated via gap junctions (connexin channels), creating an extensive interconnected network throughout the brain (Orthmann-Murphy et al., 2008). Gap junctions exchange ions and small molecules between cells (<1 kDa in size) including; inositol triphosphate (IP_3_), K^+^, Ca^2+^, ATP, glucose, glutamate, glutathione (GSH), and cyclic AMP; highlighting its signaling and buffering role within the CNS (Figure 3) (Niessen et al., 2000; Goldberg et al., 2002; Bedner et al., 2006; Lapato and Tiwari-Woodruff, 2018).

Astrocytes are involved in a number of processes associated with brain function and disease progression. Astrocytes are major secretory cells, releasing factors or transmitters (gliotransmitters) including neurotransmitters (and their precursors), modulators, peptides, hormones, trophic (growth) factors, and metabolites. Astrocyte secreted factors have been shown to be involved in synapse formation, function and plasticity, neuronal growth, differentiation and survival, as well as in the regulation of the vascular tone and blood flow in the brain. Astrocytes have also been shown to mediate synaptic pruning by phagocytosis (Chung et al., 2015; MacVicar and Newman, 2015; Verkhratsky et al., 2016).

Astrocytes become “reactive” as a response to pathological conditions or to perturbations in cellular homeostasis. Astrocyte reactivity is a loose term that primarily refers to an enlargement in the cell body and processes (hypertrophy), and an increase in the levels of the glial fibrillary acidic protein (GFAP), that relates to a pathological stimulus in the CNS. Unlike microglia, reactive astrocytes are not believed to be proliferative outside of conditions of glial scar formation, which is a specific, irreversible form of astrocyte reactivity. Reactive astrocytes contribute to the production and release of cytokines and the neuroinflammatory process during injury and neurodegeneration. Reactive astrocytes should not be confused with activated astrocytes, as the latter pertains to astrocyte responses to neurotransmission (Ben Haim et al., 2015).

Astrocytes can also be considered the master regulators of brain metabolism. In this review paper, we discuss the importance of astrocytes’ metabolism in the regulation of neurotransmitter recycling and synthesis, central carbon metabolism and bioenergetics, as well as redox homeostasis and antioxidant/xenobiotic defense. Our goal is to highlight the metabolic niche astrocytes fill within overall brain metabolism.

## Astrocytes’ Metabolism and Bioenergetics: A Glycolytic Cell

While the mass of the human brain accounts for approximately 2% of total body weight, its oxidative metabolism accounts for ∼20% of the body’s total (Rolfe and Brown, 1997; Attwell and Laughlin, 2001). Most of this oxidative energy (∼75–80%) is utilized at the synapse to maintain and restore ionic gradients, and for the uptake and recycling of neurotransmitters (Riveros et al., 1986; Wong-Riley, 1989; Attwell and Laughlin, 2001; Hyder et al., 2013; Figure 3.4). By comparison, the metabolic processes in astrocytes have been estimated to account for between 5 and 15% of the total ATP expenditure in the brain (Attwell and Laughlin, 2001; Belanger et al., 2011). Glucose is the primary energy substrate in the adult brain, however, alternate energy sources can fuel brain function. Lactate transport across the blood-brain barrier (BBB) (Figure 3.1) provides ∼8–10% of the brain’s energy requirements under basal conditions (Boumezbeur et al., 2010), and it has been estimated to supply ∼20–25% of energy during energy demanding activities (van Hall et al., 2009). As plasma lactate concentrations rise, lactate uptake in the CNS increases coinciding with a decrease in glucose uptake (Smith et al., 2003; van Hall et al., 2009). When required, an important pool of lactate is always available as the extracellular levels of lactate (0.5–1.5 mM) are similar to those of glucose (Magistretti and Allaman, 2015).

Metabolism in astrocytes and neurons is interconnected, and it has been clearly demonstrated that neurons depend upon astrocytes for a variety of metabolic processes. The *in vivo* energy demands of astrocytes can be fulfilled in the absence of oxidative phosphorylation (OXPHOS), with an observed increase in lactate production in mutants of *Cox10* (part of the cytochrome c oxidase complex and required for complex IV function), indicating that astrocytes are glycolytic (Figures 1.1, 3.2; Supplie et al., 2017). Glucose has been reported as being the preferential energy substrate of astrocytes over lactate, though glucose uptake does not match the theoretical bioenergetic output (Bouzier-Sore et al., 2006; Jakoby et al., 2014). Inside the cell, glucose is phosphorylated generating glucose-6-phosphate (P), which is subsequently converted to fructose-6P. Glucose-6P can also be used for glycogen synthesis or in the pentose phosphate pathway (PPP) (Figure 1.1), while fructose-6P can be either metabolized to pyruvate or initiate the hexosamine pathway with uridine diphosphate (UDP)-N-acetylglucosamine (GlcNAc) as its final product and mediator of protein GlcNAcylation (Goncalves et al., 2018). Downstream of glycolysis, glyceraldehyde-3P (G3P) can be metabolized for *de novo* synthesis of serine (Figure 1.1). Serine is also a precursor for the synthesis of glycine (folate cycle) and cysteine (through the trans-sulfuration pathway, TSP) (Figures 1.1, 4.4, 4.5), both of which contribute to the production of GSH (Figure 3.2), which is essential for redox buffering. Serine and glycine are major sources for the transfer of one-carbon units through folate intermediates for their use in purine and thymidylate synthesis, nicotinamide adenine dinucleotide phosphate (NADPH) production and methylation reactions (Figure 4.4; Yang and Vousden, 2016).

**FIGURE 1:**
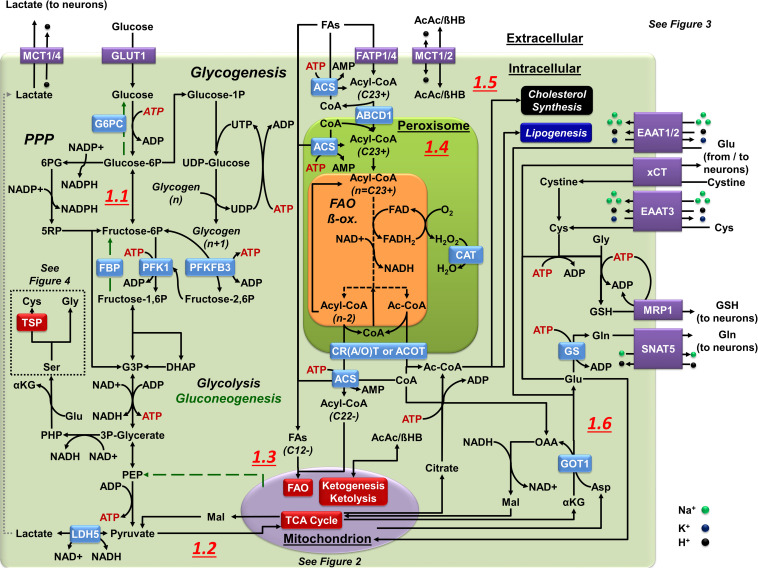
Central carbon metabolism in astrocytes. **(1.1)** In astrocytes, glucose is used via glycolysis as a principal energy source. The PPP creates redox potential by replenishing the NADPH pool. Glucose storage in glycogen is restricted or transient at most. Glucose carbons can also be used for cysteine (Cys, via TSP) and glycine (Gly) synthesis using serine (Ser) as a precursor. Gluconeogenesis (green broken arrows) is astrocyte-specific as they express FBP and G6PC but it requires metabolism of OAA to PEP in the mitochondria. **(1.2)** Pyruvate is the principal end product of glycolysis in astrocytes. Astrocytes convert most pyruvate into lactate by LDH5 to be exported to the extracellular space. Pyruvate also enters the mitochondria (see Figure 2 for details). **(1.3)** FAs are sorted by their size: 12 or less carbons can directly enter the mitochondria (SCFAs or MCFAs), 13–21 carbons (LCFAs) pass into the mitochondria through a shuttle (see Figure 2 for details), and 22+ carbons (VLCFAs) enter peroxisomes to create shorter FA products through α/β-oxidation. The esterification of FAs (ACS) limits their ability to pass through membranes, and is required for FAO. **(1.4)** Peroxisomal α/β-oxidation occurs in a similar manner to mitochondrial FAO but, without an ETC, FADH_2_ donates its electrons directly to O_2_ to create H_2_O_2_ that is neutralized by CAT. Multiple mechanisms of FA transport systems exist within peroxisomes, each with different affinities for various FA lengths. Exported FAs enter the mitochondria to complete FAO. **(1.5)** Ketone bodies in astrocytes can enter as substrates for mitochondrial metabolism (see Figure 2 for details) or can be the end product in mitochondrial metabolism that astrocytes export for other cells (neurons). **(1.6)** Astrocytes utilize glutamate (Glu) for glutamine (Gln) synthesis (Glu-Gln cycle), Cys acquisition (xCT or EAAT3), and for GSH synthesis, this later exported via (MRP1). In astrocytes, Glu is imported from the synaptic cleft by EAAT1/2 or derived from transamination of the mitochondrial TCA intermediate αKG by aspartate (Asp) amino transferases (GOT1/2). Glu can also be used to power the malate (Mal)-Asp shuttle through AGC, or enter the mitochondria as a substrate for OXPHOS (see Figure 2 for details).

Pyruvate entry into the tricarboxylic acid (TCA or Krebs) cycle (Figure 2.1) is limited by a reduced activity of pyruvate dehydrogenase (PDH) in astrocytes due to its phosphorylation via pyruvate dehydrogenase (PDK) (Bouzier-Sore et al., 2006; Halim et al., 2010; Jakoby et al., 2014), creating a surplus of pyruvate due to the high levels of glucose consumption (Figure 1.2). In contrast to astrocytes, glycolysis in neurons is restricted by the continuous degradation of 6-phosphofructo-2-kinase/fructose-2, 6-bisphosphatase-3 (PFKFB3), which catalyzes the synthesis of fructose-2,6-P an allosteric activator of phosphofructokinase 1 (PFK1) (Almeida et al., 2004; Herrero-Mendez et al., 2009). Accordingly, glucose utilization in neurons is directed to PPP in order to regenerate NADPH for the reductive recycling of GSH upon oxidative stress (Figure 3.3; Delgado-Esteban et al., 2000; Vaughn and Deshmukh, 2008; Herrero-Mendez et al., 2009; Bolanos, 2016). The astrocyte-neuron lactate shuttle (ANLS) hypothesis states that a large portion of glucose metabolism in astrocytes is directed to lactate production and this is subsequently shuttled to neurons as fuel for OXPHOS (Figures 3.2, 3.3; Pellerin and Magistretti, 1994; Magistretti and Allaman, 2015; Machler et al., 2016; Gonzalez-Gutierrez et al., 2019). This shuttle seems to involve sequestration within the endoplasmic reticulum for its delivery from perivascular endfeet to perisynaptic processes (Muller et al., 2018). Under hypoxic conditions astrocytes have the ability to upregulate glycolytic enzymes including the monocarboxylate transporter MCT4 involved in the transfer of lactate to neurons via the activation of the hypoxia-inducible factor-1α (HIF-1α) (Rosafio and Pellerin, 2014). Nitric oxide can also upregulate glucose transporter-1 (GLUT1), hexokinase-2 and MCT4 via HIF-1α (Brix et al., 2012). However, other studies have reported that selective loss of HIF-1α in astrocytes protects neurons from hypoxic injury (Vangeison et al., 2008). Recent studies also demonstrate that during energy-demanding conditions or aging neurons have the capacity to upregulate glucose metabolism, and these findings have been used to argue against the ANLS hypothesis (Figure 3.3; Patel et al., 2014; Lundgaard et al., 2015; Diaz-Garcia et al., 2017; Drulis-Fajdasz et al., 2018).

**FIGURE 2:**
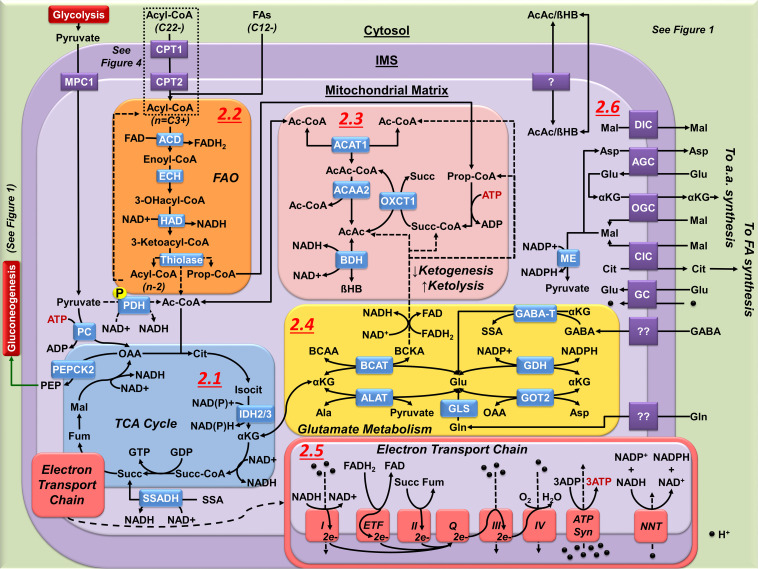
Mitochondrial metabolism in astrocytes. **(2.1)** The astrocyte TCA cycle is principally used for biosynthetic processes, not OXPHOS. Synthesis of Ac-CoA from pyruvate is limited by the phosphorylation (P) of PDH. Cataplerotic reactions of the TCA cycle are matched by anaplerotic carbon inputs. Anaplerotic inputs in astrocyte include pyruvate carboxylatyion to OAA; Ac-CoA and Succ-CoA synthesis from FAO and ketolysis; and metabolism of glutamate to αKG via transamination reactions or by the activity of GDH. Astrocyte cataplerotic outputs include PEP (gluconeogenesis) αKG, malate (Mal), and citrate (Cit). Fum, fumarate. **(2.2)** FAO produces Ac-CoA and Prop-CoA that are directed to either ketogenesis, ketolysis, or as anaplerotic input to the TCA cycle. FAO generates a high yield of NADH and FADH_2_ (*see* **2.5**). **(2.3)** Ketogenesis condenses Ac-CoA to either AcAc or βHB. FAO is the principal source of ketogenesis in astrocytes. Ketolysis reverses this process, requiring Succ-CoA from either Prop-CoA (FAO or BCAA metabolism), or the TCA cycle as a driving force. **(2.4)** Mitochondrial Glu synthesis and incorporation into the TCA cycle in astrocytes is performed by transamination (GOT2, ALAT, BCAT) or by deamination (GDH, GLS, GABA-T) of αKG allowing glutamate to act in both cataplerosis and anaplerosis. **(2.5)** The astrocyte ETC is not required for survival, and its activity has been reported to be a source of ROS due to inefficient complex activity and electron transfer. Importantly, high yield of NADH and FADH_2_ generated from FAO (see **2.2**) can increase ROS formation due to the transfer of electrons from FADH_2_ to ETF, creating a backpressure of electrons flowing from complex I. **(2.6)** While the outer mitochondrial membrane is fairly permeable, numerous transporters exist on the inner membrane to facilitate entry and exit of a number of metabolites from the mitochondrial matrix.

**FIGURE 3:**
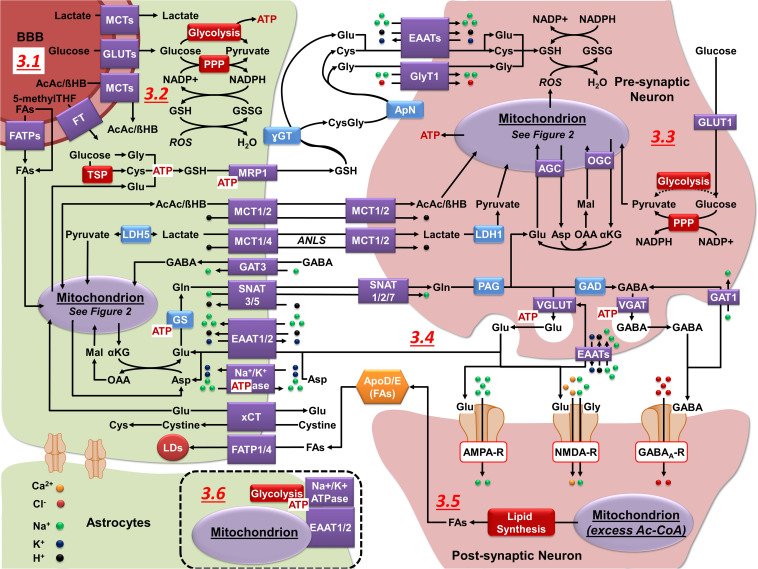
Neuronal-astrocytic metabolic cooperation. **(3.1)** GLUTs (glucose), MCTs (lactate and ketone bodies), and FATPs (FAs) facilitate the transport of carbon sources across the BBB. 5-methylTHF is the predominant form of folate in the blood and its transport across the BBB is mediated by different folate transporters (FT). **(3.2)** Astrocytes utilize glucose for the production of ATP (glycolysis), regeneration of NADPH (PPP), and to feed mitochondrial biosynthetic processes. Astrocytes synthesize and export GSH (MRP1), lactate (MCT1/4), ketone bodies (MCT1/2), and glutamine (Gln) (SNAT5) that can be utilized by neurons for energy production and synthesis of neurotransmitters. **(3.3)** Neurons utilize glucose primarily for the regeneration of NADPH (PPP), with some studies suggesting that glycolysis can be upregulated during energy-demanding conditions. Lactate and ketone bodies are used in ATP production (OXPHOS). Neurons import GSH precursors from astrocytes via EAATs and GlyT1, lactate and ketone bodies via MCT1/2, and Gln through SNAT1/2/7. **(3.4)** Upon their release into the synaptic cleft Glu and GABA neurotransmitters can be uptaken into neurons via EAATs and the GABA transporter 1 (GAT1). However, astrocytes have a higher capacity to uptake and metabolize these neuroransmitters. In astrocytes, GABA is metabolized in the mitochondria (see Figure 2.4). Glu is taken in by astrocytes (EAAT1/2), converted to Gln (GS), and exported for neuronal uptake. In the neurons, Gln is converted to Glu via PAG and then Glu can be converted to GABA via GAD. Neurotransmitters are subsequently loaded into vesicles via vesicular transporters (VGLUT or VGAT), which do not require ATP *per se*, but their activity is coupled to that of the vacuolar H^+^ ATPase. The Na^+^/K^+^ ATPase maintains the electrochemical gradient required to sustain neuronal excitability (not shown) and in astrocytes it is essential to drive neurotransmitter uptake. **(3.5)** During periods of excess Ac-CoA, lipid synthesis is initiated. Lipids are then transported to astrocytes in an apolipoprotein D/E dependent manner, taken in by FATP1/4, and transferred into LDs. **(3.6)** In astrocytes, mitochondria co-localize on the plasma membrane with EAAT1/2, Na^+^/K^+^ ATPases, and glycolytic enzymes.

**FIGURE 4:**
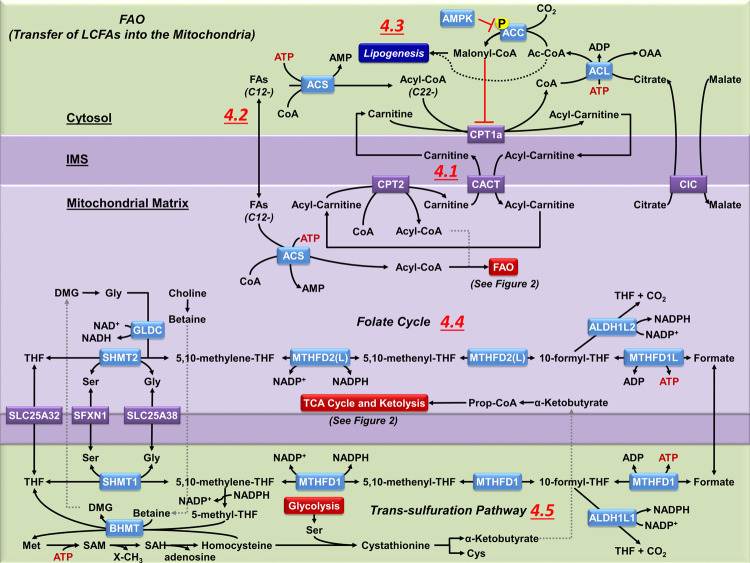
Mitochondrial carnitine shuttle, one-carbon folate metabolism and trans-sulfuration pathway. **(4.1)** Coenzyme A thioesters (Acyl-CoAs) cannot cross mitochondrial membranes. CPT1a facilitates the transfer of the acyl group from CoA to carnitine. Acyl-carnitines are permeable to the outer mitochondrial membrane, but not the inner mitochondrial membrane, requiring the transport via carnitine-acylcarnitine translocase (CACT) to reach the mitochondrial matrix. Once in the matrix, CPT2 converts the acyl-carnitine back to an acyl-CoA, which can then undergo FAO (see Figure 2.2). The released carnitine is then transported back across the inner mitochondrial membrane by CACT to be reused in the cytosol. **(4.2)** Non-esterified fatty acids of 12 or less carbons do not require the carnitine shuttle system, and can diffuse across both mitochondrial membranes. However, they still require esterification to CoA by mitochondrial ACS before they can be metabolized by FAO. **(4.3)** The carnitine shuttle system is regulated by malonyl-CoA, a precursor to FA synthesis which also inhibits CPT1a activity. Malonyl-CoA is principally produced from citrate that is broken into OAA and Ac-CoA in the cytosol by ATP citrate lyase (ACL). The released acetyl-CoA is carboxylated by ACC to malonyl-CoA. ACC is modulated by AMPK, which phosphorylates ACC and reduces its activity. **(4.4)** The folate pathway supports the transfer of one-carbon units from serine for biosynthetic processes. During this process both MTHFD2(L) and ALDH1L2 generate NADPH, which is essential for mitochondrial redox homeostasis. Despite the bidirectionality of this pathway, most of the cytosolic one-carbon units derived from serine are catabolized in the mitochondria. However, when the mitochondrial folate pathway is lost the directionality of the cytosolic one-carbon flux is reversed to compensate the synthesis of NADPH. **(4.5)** In the cytosol, MTHFD1 regenerates THF and the intermediate 5,10-methylene-THF links the folate pathway with the remethylation of homocysteine to methionine (Met) within the TSP. In the liver and kidney mitochondria, BHMT regenerates Met, linking the mitochondrial oxidation of choline to betaine with the TSP, but the presence of this pathway in brain cells has not been confirmed.

Astrocytes also store energy in the form of glycogen, which can be broken down to lactate. Yet due to the limited amount of glycogen stored in astrocytes (∼3–12 μmol per g of tissue, 1–6% of total glucose; Choi et al., 1999), its contribution to the maintenance of either neuronal or astrocyte energy-dependent processes under stress conditions is still unclear (Bak et al., 2018). There is strong evidence supporting a robust oxidative metabolic capacity in astrocytes (1/3 of total brain glucose and 1/2 of total brain lactate oxidative metabolism) (Zielke et al., 2009). Microarray and proteomic analyses have shown that most enzymes in the TCA cycle are expressed at higher levels in astrocytes than in neurons, and immuno-electron microscopy revealed that mitochondria is present at higher density in fine astrocytic processes encompassing synapses (Figure 3.6; Lovatt et al., 2007). Nevertheless, astrocytes deficient in mitochondrial respiration survive as glycolytic cells, both *in vitro* and *in vivo* (Supplie et al., 2017). What then is the physiological importance of mitochondrial metabolism in astrocytes? We next discuss the particularities of mitochondrial central carbon metabolism in these glial cells and its importance for neurotransmission and redox balance.

## Mitochondrial Metabolism in Astrocytes: Anaplerosis and Cataplerosis

Oxidative metabolism in mitochondria is primarily fueled by pyruvate, fatty acids (FAs)/ketone bodies, and glutamine/glutamate. Pyruvate generated from glycolysis is actively transported into the mitochondrial matrix via the mitochondrial pyruvate carrier (MPC1) to be subsequently decarboxylated and combined with coenzyme A (CoA) (Figure 2.1). Oxidative decarboxylation of pyruvate to acetyl-CoA (Ac-CoA) is mediated by PDH and is coupled to the reduction of nicotinamide adenine dinucleotide (NAD^+^) to NADH. Subsequently, citrate synthase catalyzes the condensation of oxaloacetate (OAA) with Ac-CoA to generate citrate. Pyruvate decarboxylation is the primary carbon source for the TCA cycle and is sufficient to sustain oxidative carbon flux during energy consumption, as the last step in the cycle regenerates OAA to be combined further with another Ac-CoA molecule generated by PDH (a closed loop) (Figure 2.1). However, TCA cycle intermediates also provide precursors for the biosynthesis of several classes of molecules. TCA intermediates leave the cycle via cataplerotic reactions linked to biosynthetic processes, while anapletoric reactions supplement carbons back into to the TCA cycle. A balance between anaplerosis and cataplerosis controls the entry and exit of carbons into the TCA cycle, and is essential for biosynthetic processes (Figure 2.1; Owen et al., 2002).

Pyruvate carboxylase (PC) is a major anaplerotic enzyme in the mitochondria that generates OAA from pyruvate in an ATP-dependent manner (Figure 2.1), replenishing carbons lost by the export of OAA, citrate, and alpha-ketoglutarate (αKG) from the TCA cycle, for gluconeogenesis, FA, and amino acid synthesis, respectively (Figures 1.2, 1.6, 2.1, 2.6). It has been estimated that 3–10% of glucose utilization in the brain is via PC, and in astrocytes, 33–50% of pyruvate entering mitochondria is utilized for anaplerotic reactions instead of producing energy (Weber and Barros, 2015). Furthermore, the metabolism of mitochondrial pyruvate through PC is reported to be 4.3-fold higher (39.3 nmol/h/10^6^ cell) than through PDH (9.1 nmol/h/10^6^ cell) (Sa et al., 2017).

In astrocytes’ gluconeogenesis, OAA is converted to phosphoenolpyruvate (PEP) in the mitochondria by phosphoenolpyruvate carboxykinase (PEPCK2), as the cytosolic activity of the cytosolic isoform (PEPCK1) has not been detected (Figure 2.1; Schmoll et al., 1995). While PEP has been shown to exit mitochondria (Garber and Ballard, 1969; Stark et al., 2009), the precise mechanism has not been determined, with the citrate/isocitrate carrier (CIC) and adenine nucleotide transporter (ANT) being proposed as likely intermediators (Stark and Kibbey, 2014). In the brain, gluconeogenesis is astrocyte-specific as they express fructose 1,6-biphosphatase (FBP), which generates fructose-6P from fructose-1,6P (Figure 1.1). Astrocytes also express glucose 6-phosphatase (G6PC) (Figure 1.1), but because they normally store glycogen, it has been suggested that gluconeogenesis in astrocytes is primarily involved in glycogenesis (Bernard-Helary et al., 2002), and that G6PC may be silent under physiological conditions becoming activated at times of stress (Ghosh et al., 2005). Gluconeogenesis in astrocytes has been demonstrated with aspartate, glutamate, alanine, and lactate as precursors and is becoming more recognized as an important alternative source of glucose for neurons during stress conditions (Yip et al., 2016).

In the brain, it has been long recognized that PC is primarily expressed in astrocytes and to a lesser extent in microglia, oligodendrocytes and ependymal cells (Shank et al., 1985; Murin et al., 2009). However, as mentioned above, PDH activity in astrocytes is reduced by its phosphorylation and thus an additional carbon source is proposed to exist to supplement Ac-CoA to the TCA cycle (Figure 2.1; Halim et al., 2010). FA oxidation (FAO) has been proposed to be the preferable pathway for Ac-CoA synthesis in astrocytes (Figures 1.4, 2.2), but its physiological implications are still unclear (Halim et al., 2010; Adina-Zada et al., 2012; Panov et al., 2014).

## Ketogenesis and Ketolysis

Ketone bodies are water-soluble molecules derived from FA metabolism in the liver, supplying energy to peripheral tissues including the brain. Ketone bodies have been shown to be produced by astrocytes as well (Blazquez et al., 1998; Cullingford et al., 1998; Guzman and Blazquez, 2001; Thevenet et al., 2016). During starvation up to ∼70% of the brain’s energy can be supplied by ketone bodies (Owen et al., 1967). Ketone bodies are produced in the mitochondria from Ac-CoA in three sequential steps mediated by: (1) Ac-CoA acetyltransferase 1 (ACAT1) also known as acetoacetyl (AcAc)-CoA thiolase or thiolase II (Ac-CoA → AcAc-CoA, a reversible reaction); (2) β-hydroxy-β-methyl glutaryl (HMG)-CoA synthase (HMCS1, AcAc-CoA → HMG-CoA); and (3) HMG-CoA lyase [HMGCL, HMG-CoA → acetoacetate (AcAc)]. However, in astrocytes, AcAc is primarily formed by the action of 3-oxo-acid-CoA transferase (OXCT1 or SCOT) or AcAc-CoA hydrolase (ACAA2) on AcAc-CoA and not by the action of HMGCL and HMCS1 (Auestad et al., 1991). AcAc is then decarboxylated to acetone or metabolized to β-hydroxybutyrate (βHB) via βHB dehydrogenase (BDH) (Figure 2.3). Ketone bodies can cross the BBB and plasma membranes via MCT1, 2 and 4 (Figures 1.5, 3.1, 3.4; Halestrap and Price, 1999; Morris, 2005). At high concentrations, ketone bodies have been proposed to support basal and activity-dependent needs of neurons and glutamatergic signaling, while decreasing glucose utilization (Lopes-Cardozo et al., 1986; Kunnecke et al., 1993; Sibson et al., 1998; Magistretti et al., 1999; Chowdhury et al., 2014; Courchesne-Loyer et al., 2017). Within the cell, ketone body transport across the mitochondrial membrane has not been fully characterized. While the mitochondrial pyruvate carrier (MPC1) has been implicated in ketone body transport, inhibition of the MPC1 does not abolish ketone body accumulation in the mitochondria, indicating that an undetermined transporter system or diffusion are involved (Figure 2.6; Paradies and Papa, 1977; Halestrap, 1978; Booth and Clark, 1981; Achanta and Rae, 2017). In mitochondria, ketolysis starts when BDH converts βHB back to acetoacetate, which is activated to AcAc-CoA by OXCT1. AcAc-CoA is then degraded by ACAT1 generating Ac-CoA that can be incorporated into the TCA cycle (Figure 2.3). Neurons, astrocytes and oligodendroglia have been shown to be fully capable of using ketone bodies as metabolic fuel *in vitro* (Chechik et al., 1987).

Ketone bodies are not toxic to the brain unless associated with ketoacidosis, a complication linked to diabetes mellitus (hyperglycemia) or alcoholism where the circulation levels of ketone bodies increase to > 10 mM. The increase in ketone bodies arises primarily from metabolism outside the brain and to date it is unclear if the toxicity is mediated by ketone body accumulation (ketosis) or acidosis (Fedorovich et al., 2018). A number of investigations have reported the beneficial effects of ketosis by regulating astrocytic metabolism, glutamate-glutamine cycle, glutamine synthetase (GS) activity, the function of vesicular glutamate transporters, excitatory amino acid transporters (EAAT), sodium-potassium adenosine triphosphatase (Na^+^/K^+^ ATPase), potassium channels (Kir4.1 and KATP), aquaporin-4, connexins, as well as on astrogliosis (Valdebenito et al., 2016; Morris et al., 2020). It has also been clearly shown that production of ketone bodies is used as a signaling mechanism to regulate food intake (Le Foll and Levin, 2016). While astrocytes exposed to ketone bodies observe significant morphological changes, these changes do not seem to correlate with gliosis or inflammation (Gzielo et al., 2019).

## FA Oxidation (FAO) in Astrocytes: The “Missing Link”

In addition to ketone bodies, FAs are also energy substrates for the brain, and unlike ketone bodies, they can diffuse across the BBB or utilize transport systems for their influx. The main source of FAs crossing into the brain are non-esterified long chain FAs (LCFAs, 13–21 carbons) complexed with albumin and circulating lipoproteins (Hamilton and Brunaldi, 2007). Transport of non-esterified FAs across the BBB is mediated by passive diffusion (FA flip-flop) or by FA transporters including FA transport proteins (FATP1 and 4, with intrinsic acyl-CoA synthetase activity) and the FA translocase (FAT/CD36) (Figure 3.1; Gnaedinger et al., 1988; Purdon et al., 1997; Hamilton and Brunaldi, 2007; Ouellet et al., 2009; Mitchell et al., 2011). The exact transport mechanisms that mediate the uptake of FAs into neurons or astrocytes are still unclear. A recent report suggests that FATP1 and 4 mediate FA uptake in glial cells. Importantly, while neurons are capable of synthesizing significant amounts of lipids during bioenergetic excess, they are reported to be dependent on an apolipoprotein E (ApoE) shuttle system to transfer them to astrocytes to avoid neurodegenerative phenotypes (Figure 3.6; Liu et al., 2015, 2017).

In the brain, FAO occurs primarily in astrocytes (Edmond et al., 1987; Auestad et al., 1991; Schonfeld and Reiser, 2013; Eraso-Pichot et al., 2018). Two recent studies comparing mitochondrial transcriptomes and proteomes between different neural cell types corroborated the increased concentration of enzymes involved in FA metabolism and transport in astrocytes compared to neurons. Specifically, enzymes associated with LCFA transport into mitochondria (carnitine palmitoyltransferases or CPTs) and enzymes that support the requirement of FAO for anaplerotic reactions and the replenishment of TCA cycle intermediates (PDK4/2 and PC) where found enriched in astrocytes (Eraso-Pichot et al., 2018; Fecher et al., 2019). FAO was also demonstrated to coexist with glycolysis and FAO enzymes seemed to be more abundant in adult vs. fetal astrocytes (Eraso-Pichot et al., 2018).

To utilize FAs metabolically (FAO), FAs must be esterified to CoA in an ATP-dependent process by acyl-CoA synthetases (ACS), a process that is often linked to, or performed shortly after, FA transport by FATPs (Figures 1.3, 4.2; Mashek et al., 2007; Anderson and Stahl, 2013). In the brain, signaling through the peroxisome proliferation activated receptor beta (PPARβ, predominantly expressed in neurons but also glia) increases the expression of ACS2 (Basu-Modak et al., 1999), in turn increasing the oxidation of exogenously supplied FAs in neurons (Marszalek et al., 2004). Astrocytes have an increased expression of both PPARα and ACS1 compared to neurons (Basu-Modak et al., 1999), which has been linked to the upregulation of genes involved in FAO and ketogenic processes (Cullingford et al., 2002).

Carnitine palmitoyltransferase 1a (CPT1a) transfers the acyl groups from esterified FAs (acyl-CoA) to L-carnitine (Figure 4.1), producing acyl-carnitine esters which are transported across the outer and inner mitochondrial membranes via the voltage-dependent anion channel (VDAC) and the carnitine/acyl-carnitine translocase (CACT) transport, respectively. Then, CPT2 converts acyl-carnitine back to acyl-CoA in the mitochondrial matrix (Figure 4.1; Houten and Wanders, 2010; Panov et al., 2014; Romano et al., 2017). While both astrocytes and oligodendrocytes uptake saturated and unsaturated FAs (Hofmann et al., 2017), CTP1a seems to be found primarily in astrocytes and neural progenitor cells, while absent in neurons, microglia, and oligodendrocytes (Jernberg et al., 2017). As such, carnitine deficiency causes a metabolic encephalopathy that is characterized by astrocytic swelling and mitochondrial expansion, corroborating that FAO is an essential metabolic component of astrocytic function (Kimura and Amemiya, 1990; Calabrese et al., 2005; Jones et al., 2010). CPT1a activity is negatively regulated by malonyl-CoA, a molecule synthesized by Ac-CoA carboxylase (ACC) from cytosolic Ac-CoA. Malonyl-CoA is also a principal component of FA synthesis, making this molecule the regulator of FAO and lipogenesis (Figure 4.3; Foster, 2012). ACC is inhibited by phosphorylation via the energy sensor adenosine monophosphate (AMP)-activated protein kinase (AMPK), which links energy deficiency with mitochondrial FAO (Figure 4.3). Recurrent low glucose exposure has been shown to activate AMPK and increase FAO dependency in astrocytes (Weightman Potter et al., 2019). In another study, astrocyte activation with the ciliary neurotrophic factor (CNTF) was also shown to activate AMPK and increases FAO and ketolysis (Escartin et al., 2007). In contrast, upon hypoxic conditions or high concentrations of FAs, ketogenesis is induced in astrocytes and this is regulated as well by AMPK signaling (Blazquez et al., 1999; Takahashi et al., 2014).

In the mitochondria, acyl-CoAs are principally broken down into Ac-CoA by FAO in a series of sequential reactions that include: (1) an initial flavin adenine dinucleotide (FAD)-dependent dehydrogenation of acyl-CoAs by acyl-CoA dehydrogenase (ACD or ACAD) that generates FADH_2_; (2) a subsequent hydration step mediated by enoyl-CoA hydratase (ECH) forming 3-hydroxy(OH)acyl-CoA; (3) generation of 3-ketoacyl-CoA via 3-OHacyl-CoA dehydrogenase (HAD) in an NAD^+^-dependent manner regenerating NADH; and (4) cleavage and release of Ac-CoA via thiolase I leaving an acyl-CoA two carbon atoms shorter that re-enters the pathway. The last three steps are carried by a heteroctamer protein complex called the mitochondrial trifunctional protein composed of alpha [HADHA (ECH/HAD)] and beta [HADHB (thiolase)] subunits (Figure 2.2; Houten and Wanders, 2010; Panov et al., 2014; Romano et al., 2017). In the brain, 95% of HADHA co-localizes with the astrocyte biomarker GFAP (Sayre et al., 2017) and ACD is also predominantly find in astrocytes (Fecher et al., 2019).

For FAs/acyl-CoAs with odd numbered carbons, FAO will yield propionyl (Prop)-CoA rather than Ac-CoA from the final carbons of the acyl-CoA. Prop-CoA can either be converted to succinyl (Succ)-CoA in an ATP-dependent manner by a series of reactions providing an anaplerotic input to the TCA cycle, or act as a co-substrate in the ketolytic conversion of AcAc-CoA to acetoacetate by OXCT1 (Figure 2.3). In the event that the β-carbon of a FA is methylated, α-oxidation occurs to remove one carbon unit as formic acid [formic acid is converted to carbon dioxide (CO_2_)], shifting the methylated carbon from the β to α position, allowing β-oxidation to resume. The steps in α-oxidation include: (1) hydroxylation of the α-carbon by phytanoyl-CoA hydroxylase (PhyH/Pahx) utilizing the conversion of αKG to succinate as a driving force; (2) decarboxylation by 2-hydroxyphytanoyl-CoA lyase (2-HPCL) releasing formyl-CoA and a fatty aldehyde; (3) oxidation of the fatty aldehyde by aldehyde dehydrogenase generating NADH and an acyl-CoA one carbon shorter than the original product (Jansen and Wanders, 2006).

A large quantity of ATP can be generated from FAO (one molecule of palmitic acid provides approximately 115 ATPs vs. 32 ATPs generated from glucose oxidation). Despite its high-energy potential, FAs have long been considered a poor energy substrate in the brain. The brain as a whole was thought to be limited in FAO capacity as thiolase I, ACD and ECH activities are 0.7, 50, and 19% that of the heart mitochondria (Yang et al., 1987), though it must be noted that glial and neuronal forms of the enzymes were not assayed independently (Figure 2.2). While there are three known carnitine palmitoyltransferase 1 (CPT1) isoforms, CPT1a has a low expression in the whole brain and is not expressed in neurons (Jernberg et al., 2017), CTP1b is not expressed in the brain (Obici et al., 2003; Lavrentyev et al., 2004), and CPT1c localizes exclusively to the endoplasmic reticulum in neurons and has not been shown to participate in FAO (Wolfgang et al., 2006; Sierra et al., 2008) but has been implicated in ROS management (Lee and Wolfgang, 2012). Lower CPT1a expression and activity restricts mitochondrial oxidation of FAs in neurons (Bird et al., 1985), except for those with 12 or fewer carbons, which can passively diffuse across the mitochondrial inner membrane (Figures 1.3, 2.2, 4.2). Yet the brain has been demonstrated to metabolize carbons from FAs *in vivo*, accounting for ∼20% of the total Ac-CoA pool when labeled octanoate is supplied to the bloodstream (Ebert et al., 2003), which occurs predominantly in astrocytes, based on comparisons of astrocytes, oligodendrocytes and neurons in primary cultures (Edmond et al., 1987).

Astrocytes have a high buffering capacity against ROS compared to neurons (Sun et al., 2006), and FAO is proposed to induce higher amounts of ROS formation. One cycle of FAO generates one molecule of FADH_2_ and NADH, increasing the total FADH_2_/NADH ratio per Ac-CoA created compared to glycolysis (Figure 2.2). When ACD transfers electrons from acyl-CoA to FAD, it is coupled to the subsequent transfer of electrons from the resultant FADH_2_ to the electron transferring flavoprotein (ETF). The follow-up transfer of electrons from ETF to ubiquinone (coenzyme Q) by ETF dehydrogenase creates a backpressure of electrons flowing through complex I (donated from NADH) and complex II (donated from succinate) to ubiquinone that enhances the probability of leakage and formation of ROS (Figure 2.5). Thus, it has been proposed that restricting FAO protects neurons against oxidative damage beyond the stages of development (Schonfeld and Reiser, 2013, 2017). In the brain, inhibition of FAO by methyl palmoxirate has been shown to reduce the concentrations of non-enzymatically oxidized metabolites derived from poly-unsaturated FAs (PUFA), indicating that ROS are being generated from FAO (Chen et al., 2014).

However, the mechanism of ROS generation from FA substrates is not universal, and depends upon the length and structure of the FA. Very long chain FAs (VLCFAs, 22 or more carbons) that are not esterified have been shown to integrate into artificial phospholipid bilayers, desorpting at slower rates than shorter FAs and disrupting membrane fluidity (Ho et al., 1995). This integration and disruption by VLCFAs has been shown to occur in the inner mitochondrial membrane, decreasing the membrane potential by protonophoric activity, and reducing ROS generated from reverse electron transport (RET) (Hein et al., 2008). The protonophoric property, and its resulting decrease in ROS from hyperpolarization, has been demonstrated in medium chain FAs (MCFAs 6–12 carbons) (Korshunov et al., 1998), LCFAs and branched chain FAs (Schonfeld and Wojtczak, 2007). This protonophoric capacity of FAs decreases as the length of the FA decreases (Schonfeld and Wojtczak, 2016), while branched FAs such as phytanic acid show an increased protonophoric activity compared to their straight chain FA counterparts (Komen et al., 2007; Schonfeld and Reiser, 2016). Additionally, application of carnitine esters as substrates demonstrated that RET was not occurring in the mitochondria, furthering that RET is not a prominent form of ROS production during FAO (Schonfeld et al., 2010).

While protonophoric properties of FAs can mitigate the effects of hyperpolarization/RET, FAs can promote ROS generated through forward electron transport and by interfering with electron transport. Isolated mitochondria increased ROS production proportionally to FA exposure in the presence of the recoupling agent carboxyatractyloside (Schonfeld and Wojtczak, 2007) and during uncoupling with CCCP (Cocco et al., 1999), indicating that ROS was being generated independently of the protonophoric effects. Interestingly, branched phytanic acid and poly unsaturated arachidonic acid demonstrated stronger ROS effects than unbranched saturated FAs in the aforementioned studies (Cocco et al., 1999; Schonfeld and Wojtczak, 2007). Mitochondria exposed to low concentrations of LCFAs were also found to generate ROS at complex III and in a membrane potential-independent manner at the ETF-ETF-oxidoreductase (Seifert et al., 2010). As described in Schonfeld and Wojtczak (2008), despite the evidence of electron transfer interference by FAs, the mechanism is not well understood.

FAs are stored within cells as energy-rich triacylglycerides localized in lipid droplets, removing excess free FAs from the cytoplasm, which are toxic and disrupt mitochondrial membrane integrity. In addition, LDs deliver FAs into mitochondria for their consumption as an alternative energy source during periods of nutrient depletion. Neurons do not typically make LDs, while astrocytes do (Schonfeld and Reiser, 2013), and it has been shown that toxic FAs produced in hyperactive neurons are transferred to astrocytic LDs where they are consumed by via mitochondrial FAO (Ioannou et al., 2019). Importantly, under nutrient deprivation conditions, the survival of astrocytes depends on LD-fueled FAO (Cabodevilla et al., 2013).

Peroxisomes are located throughout the brain but are primarily found in astrocytes and oligodendrocytes (Troffer-Charlier et al., 1998). VLCFAs cannot be transported into the mitochondria via the carnitine shuttle and thus, are broken down first by peroxisomes. As in mitochondria, FAs must be esterified to CoA before undergoing FAO in peroxisomes (Figures 1.4, 4). Because peroxisomes lack an electron transport chain (ETC), electrons from FADH_2_ are transferred directly to O_2_ creating hydrogen peroxide (H_2_O_2_) that is scavenged by catalase (CAT). Within the peroxisome, FAs are shortened to Ac-CoA, Prop-CoA, and a wide range of LCFAs, MCFAs, and short chain FAs (SCFAs, 6 carbons or less). A variety of export systems exist within the peroxisomes, each with different affinities for different lengths of FAs, including carnitine-transferases [carnitine acetyltransferases (CRATs), carnitine octanoyltransferases (CROTs)], and thiolases [acyl-CoA thioesterases (ACOTs)] (Figure 1.4; Antonenkov and Hiltunen, 2012). Additionally, peroxisomes have been shown to possess ketogenic enzymes, implicating peroxisomes as another potential source of ketone bodies and cholesterol (Hovik et al., 1991; Antonenkov et al., 2000; Olivier et al., 2000).

Dysfunction of peroxisomal processes has been linked to demyelination, oxidative stress, inflammation, cell death, and abnormalities in neuronal migration and differentiation (Trompier et al., 2014). Defects in the ATP binding cassette subfamily D member 1 (ABCD1) transporter that translocates very LCFAs into peroxisomes (Figure 1.4), leads to the accumulation of these fats in cells and tissues. VLCFAs and branched chain FAs can interfere directly with the ETC by opening the permeability transition pore, disrupting Ca^2+^ balance and depolarizing the mitochondria (Hein et al., 2008; Kruska et al., 2015; Schonfeld and Reiser, 2016). LCFAs activate PPAR transcription factors that stimulate both FAO and ketogenesis as well as mitochondrial and peroxisome biogenesis. Recent reports have also demonstrated that peroxisome biogenesis requires the generation of pre-peroxisomes from mitochondrial derived vesicles (Sugiura et al., 2017).

Previous studies have demonstrated that astrocytes have the machinery to metabolize MCFAs, LCFAs and VLCFAs as evidenced not only by the higher expression of FAO-related enzymes but also by the higher content of peroxisomes (Fecher et al., 2019; Fitzner et al., 2020). For example, the MCFA octanoate (C8) that composes 13% of the normal free fatty acid pool and can cross the BBB is avidly metabolized by astrocytes (Ebert et al., 2003). In brain slices, astrocytes have been shown to contain palmitate (C16) and oleate (C18) LCFAs (Hofmann et al., 2017).

Alterations in FA metabolism in astrocytes have been reported in different disorders. A recent report demonstrated that in a Huntington’s disease mouse model, astrocytes within the striatum consume FAs as an alternative fuel, which results in increased oxidative damage (Polyzos et al., 2019). Astrocytes carrying the ApoE4 allele, the strongest genetic risk factor for the development of Alzheimer’s disease, observe profound alterations in FA metabolism and LD formation (Farmer et al., 2019). Stimulation of FAO protects against ischemic injury as well (Sayre et al., 2017). Pharmacological modulation of FAO has been suggested as a therapeutic approach against glioblastoma cells (Lin et al., 2017). Many chemical compounds are considered to be inhibitors or activators of FAO enzymes. However, the current knowledge on their specificities, potencies and metabolic impacts is limited and controversial. For example, at high concentrations, etomoxir, the inhibitor of CPT1a, has off-target effects linked to the inhibition of the ETC (Yao et al., 2018). Other FAO modulators include the CPT1a inhibitor oxfenicine and PPARs agonists/antagonists. Accordingly, the impairment in FAO observed in astrocytes derived from induced pluripotent stem cells isolated from Alzheimer’s disease patients is corrected by PPARβ/δ agonists (Konttinen et al., 2019). The specificity of the metabolic modifiers ranolazine (Ranexa) and trimetazidine, initially described as partial FAO inhibitors that target the HAD (or 3-KAT) activity of the trifunctional protein HADHA, has been recently questioned (Ma et al., 2020).

## Mitochondrial Metabolism in Astrocytes and Neurotransmitter Homeostasis

Astrocyte processes extending to synaptic clefts play an important role in regulating synaptic transmission, particularly glutamatergic signaling. After its vesicle-mediated release, the excitatory neurotransmitter glutamate is taken from the synaptic cleft by astrocytes in a Na^+^ dependent manner via EAAT1 (glutamate aspartate transporter, GLAST) or EAAT2 (glutamate transporter 1, GLT-1) (Figure 3.4). In the cytosol, glutamate is then converted into non-excitatory glutamine via GS, and transferred back to neurons via the Na^+^-coupled neutral amino acid transporters (SNATs) to be converted back into glutamate in neurons via phosphate-activated glutaminase (PAG) (Figure 3.4; Norenberg and Martinez-Hernandez, 1979; Anderson and Swanson, 2000; Schousboe et al., 2013; Leke and Schousboe, 2016). In astrocytes, despite the presence of a weak mitochondrial targeting sequence, GS is localized in the cytosol because the mitochondrial membrane potential is not negative enough to drive GS into the mitochondria (Figures 1.6, 3.2; Matthews et al., 2010). Neurons lack PC activity (Yu et al., 1983), restricting their ability to replenish TCA intermediates lost during neurotransmitter release (anaplerotic reaction). Thus, the glutamate-glutamine cycle between neurons and astrocytes, not only terminates/modulates glutamatergic signaling, preventing excitotoxicity triggered by excessive or prolonged exposure to glutamate, but it also replenishes the neurotransmitter pool in neurons (Figure 3.4; Belanger et al., 2011).

Not all the glutamate taken from the synaptic cleft by astrocytes becomes glutamine. There is evidence that a significant portion is incorporated into the TCA cycle via oxidative metabolism (Figure 2.4). Glutamate transport into the mitochondria is mediated via the aspartate/glutamate carrier antiporter (AGC or Aralar 1) or via the glutamate carrier (GC1 or GC2), a symporter for glutamate and H^+^. Controversy still exists about the contribution of AGC to the uptake of glutamate in astrocytic mitochondria, as it has been shown to be primarily expressed in neurons. However, while the AGC activity in astrocytes corresponds only to 7% of the total brain AGC activity, it is calculated to be twice the minimum required for glutamate production and degradation (Figure 2.6; Hertz, 2011). AGC is also central to the malate-aspartate shuttle (Figure 1.6), a system that allows the transfer of NADH electrons generated in the cytosol, from processes such as glycolysis, to the mitochondria (Amoedo et al., 2016). On the other hand, knockdown of GC1 suggests that this carrier is the main gate for net glutamate entry into mitochondria for oxidative metabolism in astrocytes (Figure 2.6; Goubert et al., 2017).

In the mitochondria, glutamate is metabolized to αKG by transamination via aspartate aminotransferase (GOT2), and to a lesser extent by alanine (ALAT) and branched chain aminotransferases (BCAT) (Figure 2.4). In addition, αKG can also be synthesized from glutamate via glutamate dehydrogenase (GDH), which is also a source of NADPH. While glutamate transamination via GOT2 generates a truncated TCA cycle due to the requirement of OAA as a co-substrate, GDH-mediated dehydrogenation can serve as an anaplerotic reaction to replenish TCA intermediates (Figures 2.1, 2.4; Sonnewald et al., 1993; Olsen and Sonnewald, 2015; McKenna et al., 2016). Astrocytes deficient in GDH increase their reliance on GOT2 and their glycolytic input to PC, which is paralleled by an accumulation of intracellular glutamate, underlying the importance of GDH in anaplerosis (Nissen et al., 2015; Pajecka et al., 2015). It should be noted that humans, unlike most other animals including mice and rats, have an additional GDH2 isoform that is expressed solely in astrocytes. Expression of GDH2 in rat cortical astrocytes increased glutamate uptake and metabolism, reducing their dependence on glucose and increasing their capacity to utilize branched chain amino acids (BCAA) (Nissen et al., 2017). Importantly, glutamate to glutamine metabolism is not determined by the extracellular glutamate concentration. However, the entry of glutamate into the TCA cycle seems to be triggered by high extracellular glutamate concentrations (high μM) (McKenna et al., 1996).

The homeostasis of the inhibitory neurotransmitter γ-aminobutyric acid (GABA) is similarly linked to the glutamate-glutamine cycle. GABA is synthesized in neurons from the decarboxylation of glutamate by glutamate decarboxylase (GAD). After its vesicular release, GABA is uptaken by astrocytes through the GABA transporter (GAT3) (Figure 3.4). GABA is then metabolized by GABA transaminase (GABA-T) with αKG as cofactor to produce succinate semialdehyde (SSA) and glutamate (Figure 2.4). SSA is metabolized to succinate via SSA-dehydrogenase (SSADH), which can then be incorporated back into the TCA cycle. This anaplerotic addition of succinate compensates the cataplerotic loss of carbons from the generation of glutamate by the GABA-T (Figure 2.1; Schousboe et al., 2013).

In addition to metabolism, evidence indicates that astrocyte mitochondrial bioenergetics are important for the regulation of neurotransmitter balance. Both glutamate and GABA uptake systems in astrocytes are driven by Na^+^ gradients that are established by the electrogenic action of the Na^+^/K^+^ ATPase (Figure 3.4). PC and GS activities also require ATP as well, and are critical enzymes in replenishing neuronal glutamine/glutamate (FIgures 2.1, 3.4). Importantly, it has been demonstrated that mitochondria co-localize with EAAT1/2 and glycolytic enzymes on the plasma membrane of astrocytes (Genda et al., 2011; Bauer et al., 2012), and that glutamate transporters physically interact with the Na^+^/K^+^ ATPase (Figure 3.6; Rose et al., 2009), suggesting that while mitochondrial-derived ATP is not required for astrocyte survival (Supplie et al., 2017), functional mitochondrial metabolism is important for neurotransmitter uptake and homeostasis (Figures 3.2, 3.4, 3.6). In fact, astrocyte processes enveloping synaptic terminals contain abundant mitochondria (Jackson et al., 2014; Stephen et al., 2015; Jackson and Robinson, 2018), and inhibition of mitochondrial function by fluorocitrate in astrocytes increases glutamate excitotoxicity in co-cultures with neurons (Voloboueva et al., 2007).

## Mitochondrial Metabolism in Astrocytes and Redox Balance

Astrocytes contain higher levels of endogenous antioxidants and antioxidant systems being more resistant to oxidative stress than neurons, which is explained by the activation of the antioxidant response via the nuclear factor erythroid-2-related factor 2 (Nrf2) transcription factor (Shih et al., 2003; Jimenez-Blasco et al., 2015). Astrocytes also have higher levels of NADPH and G6PD (glucose 6-phosphate dehydrogenase) glucose 6-phosphate dehydrogenase (G6PD) (Garcia-Nogales et al., 2003). In the mitochondria, superoxide anion (O_2_^•–^) is generally considered to be generated in the matrix by electron leakage from complex I (Grivennikova and Vinogradov, 2006), and in both the matrix and the intermembrane space (IMS) by electron leakage from complex III (Chen et al., 2003). O_2_^•–^ generated in the matrix and the IMS has been proposed to be released to the cytosol via the mitochondrial permeability transition pore (mPTP) (Wang et al., 2008) and the VDAC (Han et al., 2003), respectively. However, at least 10 different additional sites for O_2_^•–^/H_2_O_2_ production in the mitochondria have been described. The relative and absolute contribution of specific sites in the mitochondria to the production of ROS seems to depend on the availability of the substrate being oxidized (Cardoso et al., 2012; Quinlan et al., 2013). Of importance, one ROS can lead to the generation of distinct more reactive species. As such, O_2_^•–^ can be dismutated in an enzymatic (superoxide dismutases [SODs]) or non-enzymatic manner to H_2_O_2_. While H_2_O_2_ is thought to diffuse across membranes, it has also been reported that it can be transported by aquaporins (Bienert et al., 2006, 2007). Surprisingly, astrocytes have less efficient mitochondrial respiration and increased ROS formation when compared to neurons, in part due to a lower incorporation of complex I into supercomplexes in astrocytes caused by lower expression of the NADH-ubiquinone oxidoreductase core subunit S1 (NDUFS1) (Lopez-Fabuel et al., 2016).

The physiological role of increased mitochondrial ROS production in astrocytes had not been explored before, but an elegant recent investigation using mitochondria-targeted antioxidants demonstrated that high ROS production in astrocytes regulates GSH metabolism via Nrf2, and conversely, represses glucose metabolism via the PPP to decrease NADPH-dependent ROS production by NADPH-dependent oxidases (Nox1 and 2), which together modulate the redox status and survival of neurons (Vicente-Gutierrez et al., 2019). Interestingly, glutamate (N-methyl-d-aspartate) receptors (NMDA-R) couple neurotransmission to Nrf2 activation, where NMDA-R signaling in astrocytes promotes Nrf2 phosphorylation by cyclin-dependent kinase 5 (Cdk5) and its translocation to the nucleus, a novel mechanisms of intercellular feedback communication from neurons to astrocytes (Jimenez-Blasco et al., 2015).

While both neurons and astrocytes can synthesize GSH, astrocytes have been reported to protect neurons via regulation of GSH metabolism (Chen et al., 2001; Shih et al., 2003). This protective effect is thought to be dependent on the supply of cysteine from astrocytes as a precursor for *de novo* GSH synthesis (Dringen et al., 1999; Wang and Cynader, 2000), and in part due to neurons being unable to uptake significant amounts of extracellular cystine (Sagara et al., 1993; Kranich et al., 1996). GSH has been proposed to be released from astrocytes via the ATP-binding cassette transporters subfamily C member 1 transporter (ABCC1, or multidrug-resistance-associated protein 1 [MRP1]) (Figures 1.6, 3.2; Hirrlinger et al., 2002; Hirrlinger and Dringen, 2005). Indeed, inhibition of MRP1 has been reported to reduce the extracellular accumulation of GSH in cultured astrocytes (Hirrlinger and Dringen, 2005). We have failed to observe a reciprocal accumulation of GSH in astrocytess when MRP1 activity is reduced (*unpublished data*), but this effect might be ascribed to feedback inhibition of GSH synthesis by GSH.

Once released, GSH is degraded by the γ-glutamyl transpeptidase (γGT) to produce l-cysteine-l-glycine (CysGly) (Dringen et al., 1997). CysGly is cleaved further by the neuronal aminopeptidase N (ApN) into the amino acids glycine and cysteine that are taken up by neurons for *de novo* GSH synthesis via the Na^+^-Cl^–^-dependent glycine transporter 1 (GlyT1) and EAAT3, respectively (Figures 3.2, 3.3; Dringen et al., 1999, 2001; Aoyama et al., 2008; Belanger et al., 2011). The export of GSH should imply a loss of carbons that must be replenished in astrocytes to maintain cellular redox homeostasis. Cysteine acquisition in astrocytes is mediated by the Na^+^-dependent X_AG_-/EAAT3 and -independent X_c_-/xCT uptake systems, but their exact contribution is still under debate (Shanker et al., 2001; Seib et al., 2011). *De novo* synthesis via the TSP pathway is also an important source for cysteine in astrocytes, and was shown to be upregulated and utilized during oxidative stress (Figures 3.2, 4.5; Vitvitsky et al., 2006). In most cells, cysteine is considered the limiting substrate for GSH synthesis. However, glutamate might also be considered a limiting in astrocytes as it is metabolized to glutamine (Figure 1.6). Because glutamate synthesis depends on mitochondrial anaplerotic metabolism, mitochondrial function might as well be essential for GSH homeostasis (Figure 2.1, 2.4). Interestingly, GSH depletion upregulates mitochondrial activity and expression of complex I in astrocytes (Vasquez et al., 2001), but the exact mechanisms that regulate this phenomenon are still unclear.

Central carbon metabolism in the mitochondria is also important for the maintenance of redox balance as it fuels the regeneration of NADPH. In addition to the anaplerotic activity of GDH mentioned above (Figure 2.4), within the mitochondria, the malic enzyme (ME) and isocitrate dehydrogenase isoform 2 (IDH2) regenerate NADPH coupled to the formation of OAA and αKG, respectively (Figures 2.1, 2.6). While higher levels of mitochondrial IDH activity have been reported in astrocytes (Minich et al., 2003), conflicting results exists in regards to the activity levels of mitochondrial ME (McKenna et al., 1995; Vogel et al., 1998). Metabolic flux analysis has also demonstrated that transfer of H from NADH via nicotinamide nucleotide transhydrogenase (NNT) contributes to the regeneration of the mitochondrial NADPH pool (Figure 2.5; Lewis et al., 2014), which is highly expressed in the brain (Arkblad et al., 2002). A comparative analysis demonstrated that these three metabolic pathways actively contribute to NADPH replenishment in brain mitochondria (Vogel et al., 1999), but other studies have reported a predominant role for the NNT pathway, particularly under metabolic stress (Lopert and Patel, 2014).

Folate metabolism supports one-carbon metabolism that serves to activate and transfer one-carbon units for biosynthetic processes. Mammals lack the capacity for folate synthesis whose requirements are largely met through dietary sources. Folate is not biologically active, but it is readily reduced in the intestinal mucosa or liver and converted into tetrahydrofolate (THF) molecules, such as 5-methyl-THF and 5-formy-THF. 5-methylTHF is the predominant form of folate in the blood and its transport in the brain across the BBB is mediated by the folate receptor alpha (FRα/FOLR1), the H^+^-coupled folate transporter (PCFT/SLC46A1), and the reduced folate carrier (RFC/SLC19A1), but whether the same mechanisms mediate folate transport in neural cells including astrocytes is unclear (Figure 3.1; Alam et al., 2020). Furthermore, because there is no dihydrofolate reductase in the brain to catalyze the formation of 5-methyl-THF (Wu and Pardridge, 1999), 5-methylTHF rather than folate is present in the CSF. Carrier-mediated energy-dependent transport of 5-methyl- and 5-formyl-THF has been described in astrocytes (Cai and Horne, 2003).

While serine can be acquired from the diet, central carbon metabolism makes substantial contribution to serine availability and as a consequence to folate metabolism in both the cytosol and the mitochondria (Figure 4.4). THF and serine are transported into the mitochondria via SLC25A32 (Lawrence et al., 2011) and sideroflexin-1 (SFXN1) (Kory et al., 2018), respectively. Mitochondrial serine hydroxymethyltransferase 2 (SHMT2) transfers the β-carbon of serine to THF, generating 5,10-methylene-THF that can also be produced from glycine by the activity of glycine decarboxylase (GLDC), an abundant enzyme in astrocytes (Figure 4.4; Li et al., 2018). GLDC deficiency induces neural tube defects and hydrocephaly (Pai et al., 2015; Leung et al., 2017; Santos et al., 2020). The bifunctional enzyme methylenetetrahydrofolate dehydrogenase 2 (MTHFD2) or MTHFD2-like (MTHFD2L) then catalyzes the sequential production of 5,10-methenyl-THF and 10-formyl-THF. Finally, 10-formyl-THF is cleaved by MTHFD1L to generate formate, which can be exported to the cytosol (Figure 4.4). 10-formyl-THF can also be metabolized by 10-formyl-THF dehydrogenase (ALDH1L2). During this process both MTHFD2(L) and ALDH1L2 generate NADPH (Yang and Vousden, 2016; Figure 4.4). As such, knockdown of MTHFD2 decreases cellular NADPH/NADP^+^ and GSH/GSH disulfide (GSSG) ratios, and sensitizes cells to oxidative stress (Fan et al., 2014). The cytosolic counterpart of ALDH1L2, ALDH1L1 is expressed abundantly in astrocytes (Neymeyer et al., 1997; Foo and Dougherty, 2013). ALDH1L2 mutations seem to induce astrocytic stress (Sarret et al., 2019). Interestingly, complex I deficiency has been shown to decrease NADPH synthesis via ALDH1L2 due to the impaired generation of NAD^+^ (Balsa et al., 2020). Despite the bidirectionality of this pathway, most of the cytosolic one-carbon units derived from serine are catabolized in the mitochondria, as this seems to be favored by the more oxidative mitochondrial redox potential, when compared to the cytosol. However, when the mitochondrial folate pathway is lost the directionality of the cytosolic one-carbon flux is reversed to compensate the synthesis of NADPH (Ducker et al., 2016).

In the cytosol, the trifunctional enzyme MTHFD1 regenerates THF and the intermediate 5,10-methylene-THF links the folate pathway and thus, central carbon metabolism, with the remethylation of homocysteine within the TSP. As mentioned before the TSP is an important source for cysteine in astrocytes (Figures 3.2, 4.5; Vitvitsky et al., 2006). While remethylation of homocysteine to methionine can be accomplished by the activity of methionine synthase, in the liver and kidney mitochondria, betaine-homocysteine S-methyltransferase (BHMT) also produces methionine from homocysteine, linking the mitochondrial oxidation of choline to betaine with the TSP (Figure 4.5). Brain genetic variants of BHMT have been linked to schizophrenia (Ohnishi et al., 2019). However, the presence of BHMT and its significance to the remethylation of homocysteine hasn’t been confirmed in astrocytes (Kempson et al., 2014).

## Conclusion and Perspectives

The scientific process has been aptly described as blind people attempting to describe an elephant while only focusing on particular parts of the animal. Each person is contextually correct, but only when the data is combined can we achieve an accurate understanding of the whole. While there are far more parts of the proverbial elephant to explore to complete the picture, the recent evidence indicates that we must adjust our current understanding of brain metabolism, particularly as it pertains to astrocytes and neurons.

Current research indicates that metabolism in the brain extends far beyond glucose. FAs, and their derived ketone bodies, are important sources of bioenergetic potential for the brain, particularly during development, as shown by the experiments *in vivo* and *in vitro* that follow its oxidation and integration into biomolecules. Glutamate is more than just a neurotransmitter kept in strict stoichiometry by the glutamate-glutamine cycle; it is a resource the brain uses for amino acid synthesis by transamination, for redox balance by generation of NADPH, and for anaplerosis to maintain the TCA cycle.

The brain is far from a homogeneous body of cells, and the literature paints a clear picture of metabolic specialization and intracellular cooperation/dependence. In this review, we have centered our attention to astrocytic mitochondrial metabolism and its role in the regulation of brain bioenergetics, neurotransmission and redox balance via neuronal-astrocyte metabolic interactions. Astrocytes have been demonstrated to be functional without OXPHOS *in vivo*, to provide a GSH-dependent protective effect to neurons, to function as neutralizing and cycling agents of glutamatergic/GABAergic signaling, and to be synthesizers/exporters of OXPHOS bioenergetic molecules (lactate/ketone bodies). In contrast, neurons have a metabolic specialization toward OXPHOS, spurning FAO, restricting glucose oxidation while maintaining the PPP for redox homeostasis, and limiting their ability to conduct mitochondrial biosynthesis due to a lack of pyruvate carboxylase for anaplerosis. Together, neurons shoulder the bioenergetic burden of neurotransmission by controlled alterations in cell potential, while astrocytes conduct biosynthesis and high ROS oxidative processes to supplement neuronal functions.

The key organelle in this differential specialization is the mitochondria. While the broad mitochondrial role in OXPHOS is well known in mammalian cells, specialization is evident within neurons and astrocytes. Astrocytes restrict the conversion of pyruvate to Ac-CoA by their phosphorylation of PDH, limiting their utilization of glucose for OXPHOS but instead directing these carbons to biosynthesis through the TCA cycle by PC. Additionally, the efficiency of the astrocyte ETC is far less than neurons, resulting in higher ROS generation in astrocyte compared to neurons. Astrocytic mitochondria have been demonstrated to utilize FAO for the production of ketone bodies, a process that has not been shown to be significant in neurons. Further, evidence shows that astrocytes localize mitochondria to endfeet processes in conjunction with glutamate transporters and enzymes related to glycolysis. Coupled with the evidence that OXPHOS is not required in astrocytes *in vivo*, this paints a unique picture for astrocyte mitochondria compared to the prototypical mammalian cell. It is a mitochondria specializing in synthesis at the expense of OXPHOS, while the neuronal mitochondria specializes in OXPHOS at the expense of synthesis.

## Author Contributions

JR and CB contributed to the first draft and figures. AP, MP, and RF contributed to the edition of the final version of the manuscript and figures. All authors contributed to the article and approved the submitted version.

## Conflict of Interest

The authors declare that the research was conducted in the absence of any commercial or financial relationships that could be construed as a potential conflict of interest.

## Abbreviations

ABCC1/MRP1: adenosine-triphosphate-binding cassette transporter subfamily C member 1 or multidrug-resistance-associated protein 1
ABCD1: adenosine triphosphate binding cassette subfamily D member 1
AcAc: acetoacetate
Ac-CoA: acetyl-CoA
AcAc-CoA: acetoacetyl-CoA
ACAA2: acetoacetyl-CoA hydrolase
ACAT1: acetyl-CoA C-acetyltransferase, acetoacetyl-CoA thiolase II
ACC: acetyl-CoA carboxylase
ACD: acyl-CoA dehydrogenase or ACAD
ACS: acyl-CoA synthetase
ACOT: acyl-CoA thioesterases
ACL: ATP citrate lyase
ADP: adenosine diphosphate
AGC: aspartate/glutamate carrier antiporter or Aralar 1
ALAT: alanine aminotransferase
ALDH1L: 10-formyl-THF dehydrogenase
AMP: adenosine monophosphate
AMPA-R: α-amino -3-hydroxy-5-methyl-4-isoxazolepropionic acid receptor
AMPK: adenosine monophosphate activated protein kinase
ANLS: astrocyte-neuron lactate shuttle
ANT: adenine nucleotide transporter
ApN: aminopeptidase N
ApoE/D: apolipoprotein E/D
ATP: adenosine triphosphate
BBB: blood-brain barrier
BCAA: branched chain amino acids
BCAT: branched chain aminotransferases
BDH: β-hydroxybutyrate dehydrogenase
BHMT: betaine-homocysteine S-methyltransferase
CACT: carnitine/acylcarnitine translocase
cAMP: cyclic adenosine monophosphate
CAT: catalase
CACT: carnitine-acylcarnitine translocase
CIC: citrate/isocitrate carrier
Cdk5: cyclin-dependent kinase 5
CNS: central nervous system
CNTF: ciliary neurotrophic factor
CoA: coenzyme A
Cox10: cytochrome C oxidase assembly factor heme A
CPT: carnitine palmitoyltransferase
CRAT: carnitine acetyltransferase
CROT: carnitine octanoyltransferase
CysGly: l-cysteine -l-glycine
DHAP: dehydroacetone phosphate
DIC: dicarboxylate carrier
DMG: dimethylglycine
EAAT: excitatory amino acid transporter
ECH: enoyl-CoA hydratase
ETC: electron transport chain
ETF: electron transferring flavoprotein
FA: fatty acid
FAD^+^(FADH_2_): flavin adenine dinucleotide
FAO: fatty acid oxidation
FAT/CD36: fatty acid translocase
FATP: fatty acid transport protein
FBP: fructose 1,6-biphosphatase
FR α: folate receptor α or FOLR1
FT: folate transporters
GABA: γ-aminobutyric acid
GABA-T: γ-aminobutyric acid transaminase
GAD: glutamate decarboxylase
GAT: γ-aminobutyric acid transporter
GC1/2: glutamate carrier or SLC25A18/22
GDH: glutamate dehydrogenase
GFAP: glial fibrillary acidic protein
GLAST: glutamate aspartate transporter or excitatory amino acid transporter 1
GlcNAc: N-acetylglucosamine
GLDC: glycine decarboxylase
GLS: glutaminase
GLT-1: glutamate transporter 1 or excitatory amino acid transporter 2
GLUT: glucose transporter
GlyT1: sodium-chloride-dependent glycine transporter 1
GOT1: cytosolic aspartate aminotransferase
GOT2: mitochondrial aspartate aminotransferase
GS: glutamine synthetase
GSH: glutathione
GSSG: glutathione disulfide (oxidized)
G3P: glyceraldehyde 3-phosphate
G6PC: glucose 6-phosphatase
G6PD: glucose 6-phosphate dehydrogenase
γ GT: γ-glutamyl transpeptidase
HAD: 3-hydroxyacyl-Coenzyme A dehydrogenase
HADHA/B: hydroxyacyl-CoA dehydrogenase trifunctional multienzyme complex subunit α / β
(β HB: (β -hydroxybutyrate
HIF-1(α: hypoxia-inducible factor-1(α
HMCS1: (β -hydroxy-(β -methyl glutaryl-CoA synthase
HMGCL: (β -hydroxy-(β -methyl glutaryl -CoA lyase
2-HPCL: 2-hydroxyphytanoyl-CoA lyase
H_2_O_2_: hydrogen peroxide
IDH: isocitrate dehydrogenase
IMS: mitochondrial permeability transition pore
IP_3_: inositol triphosphate
(KG: (α -ketoglutarate
LCFAs: long chain fatty acid
LD: lipid droplet
LDH: lactate dehydrogenase
MCFAs: medium chain fatty acids
MCT: monocarboxylate transporter
ME: malic enzyme
MPC1: mitochondrial pyruvate carrier
MTHFD(L): methylenetetrahydrofolate dehydrogenase 2 (like)
mPTP: mitochondrial permeability transition pore
Na^+^/K^+^: ATPase sodium-potassium adenosine triphosphatase
NAD^+^(NADH): nicotinamide adenine dinucleotide
NADP^+^(NADPH): nicotinamide adenine dinucleotide phosphate
NDUFS1: nicotinamide adenine dinucleotide ubiquinone oxidoreductase core subunit S1
NMDA-R: N-methyl -d-aspartate receptors
NNT: nicotinamide nucleotide transhydrogenase
Nox: NADPH-dependent oxidases
Nrf2: nuclear factor erythroid-2-related factor 2
OAA: oxaloacetate
OGC: 2-oxoglutarate/malate carrier
3-OHacyl-CoA: 3-hydroxyacyl-Coenzyme A
OXCT1: 3-oxo-acid-CoA transferase
OXPHOS: oxidative phosphorylation
O_2_^•–^: superoxide anion
P: phosphate
PAG: phosphate-activated glutaminase
PC: pyruvate carboxylase
PCFT: proton-coupled folate transporter or SLC46A1
PDH: pyruvate dehydrogenase
PDK: pyruvate dehydrogenase kinase
PEP: phosphoenolpyruvate
PEPCK: phosphoenolpyruvate carboxykinase
PFK1: phosphofructokinase 1
PFKFB3: 6-phosphofructo-2-kinase/fructose-2,6-bisphosphatase-3
6PG: 6-phosphogluconate
PhyH/Pahx: phytanoyl-CoA hydroxylase
PPAR: peroxisome proliferation activated receptor
PPP: pentose phosphate pathway
Prop-CoA: propionyl-CoA
PUFA: poly-unsaturated fatty acid
Q: ubiquinone
Redox: reduction-oxidation
RET: reverse electron transfer
RFC: reduced folate carrier or SLC9A1
ROS: reactive oxygen species
5RP: ribulose-5 phosphate
SAH: S-adenosylhomocysteine
SAM: S-adenosylmethionine
SCFAs: short chain fatty acids
SFXN1: sideroflexin 1 or mitochondrial serine transporter
SHMT: serine hydroxymethyltransferase
SNAT: sodium-coupled neutral amino acid transporter
SSA: succinate semialdehyde
SSADH: succinate semialdehyde dehydrogenase
SOD: superoxide dismutase
Succ-CoA: succinyl-CoA
TCA: cycle tricarboxylic acid or Krebs cycle
THF: tetrahydrofolate
TSP: trans-sulfuration pathway
UDP: uridine diphosphate
UTP: uridine triphosphate
VDAC: voltage-dependent anion channel
VGAT: vesicular GABA transporter
VGLUT: vesicular glutamate transporter
VLCFA: very long chain fatty acid
XAG-/EAAT3: aspartate-glutamate transporter
Xc-/xCT: cystine-glutamate exchanger
X-CH_3_: Methylated substrate.

## References

[B1] Achanta L. B. Rae C. D. (2017). beta-Hydroxybutyrate in the brain: one molecule, multiple mechanisms. *Neurochem. Res.* 42 35–49. 10.1007/s11064-016-2099-2 27826689

[B2] Adina-Zada A. Zeczycki T. N. Attwood P. V. (2012). Regulation of the structure and activity of pyruvate carboxylase by acetyl CoA. *Arch. Biochem. Biophys.* 519 118–130. 10.1016/j.abb.2011.11.015 22120519 PMC3293938

[B3] Alam C. Kondo M. O’connor D. L. Bendayan R. (2020). Clinical implications of folate transport in the central nervous system. *Trends Pharmacol. Sci.* 41 349–361. 10.1016/j.tips.2020.02.004 32200980

[B4] Almeida A. Moncada S. Bolanos J. P. (2004). Nitric oxide switches on glycolysis through the AMP protein kinase and 6-phosphofructo-2-kinase pathway. *Nat. Cell Biol.* 6 45–51. 10.1038/ncb1080 14688792

[B5] Amoedo N. D. Punzi G. Obre E. Lacombe D. De Grassi A. Pierri C. L. (2016). AGC1/2, the mitochondrial aspartate-glutamate carriers. *Biochim. Biophys. Acta* 1863 2394–2412. 10.1016/j.bbamcr.2016.04.011 27132995

[B6] Anderson C. M. Stahl A. (2013). SLC27 fatty acid transport proteins. *Mol. Aspects Med.* 34 516–528. 10.1016/j.mam.2012.07.010 23506886 PMC3602789

[B7] Anderson C. M. Swanson R. A. (2000). Astrocyte glutamate transport: review of properties, regulation, and physiological functions. *Glia* 32 1–14. 10.1002/1098-1136(200010)32:1<1::aid-glia10>3.0.co;2-w 10975906

[B8] Antonenkov V. D. Croes K. Waelkens E. Van Veldhoven P. P. Mannaerts G. P. (2000). Identification, purification and characterization of an acetoacetyl-CoA thiolase from rat liver peroxisomes. *Eur. J. Biochem.* 267 2981–2990. 10.1046/j.1432-1033.2000.014.x 10806397

[B9] Antonenkov V. D. Hiltunen J. K. (2012). Transfer of metabolites across the peroxisomal membrane. *Biochim. Biophys. Acta* 1822 1374–1386. 10.1016/j.bbadis.2011.12.011 22206997

[B10] Aoyama K. Watabe M. Nakaki T. (2008). Regulation of neuronal glutathione synthesis. *J. Pharmacol. Sci.* 108 227–238. 10.1254/jphs.08r01cr 19008644

[B11] Arkblad E. L. Egorov M. Shakhparonov M. Romanova L. Polzikov M. Rydstrom J. (2002). Expression of proton-pumping nicotinamide nucleotide transhydrogenase in mouse, human brain and C elegans. *Comp. Biochem. Physiol. B Biochem. Mol. Biol.* 133 13–21. 10.1016/s1096-4959(02)00107-0 12223207

[B12] Attwell D. Laughlin S. B. (2001). An energy budget for signaling in the grey matter of the brain. *J. Cereb. Blood Flow Metab.* 21 1133–1145. 10.1097/00004647-200110000-00001 11598490

[B13] Auestad N. Korsak R. A. Morrow J. W. Edmond J. (1991). Fatty acid oxidation and ketogenesis by astrocytes in primary culture. *J. Neurochem.* 56 1376–1386. 10.1111/j.1471-4159.1991.tb11435.x 2002348

[B14] Bak L. K. Walls A. B. Schousboe A. Waagepetersen H. S. (2018). Astrocytic glycogen metabolism in the healthy and diseased brain. *J. Biol. Chem.* 293 7108–7116. 10.1074/jbc.r117.803239 29572349 PMC5950001

[B15] Balsa E. Perry E. A. Bennett C. F. Jedrychowski M. Gygi S. P. Doench J. G. (2020). Defective NADPH production in mitochondrial disease complex I causes inflammation and cell death. *Nat. Commun.* 11:2714. 10.1038/s41467-020-16423-1 PMC7264245 32483148

[B16] Basu-Modak S. Braissant O. Escher P. Desvergne B. Honegger P. Wahli W. (1999). Peroxisome proliferator-activated receptor beta regulates acyl-CoA synthetase 2 in reaggregated rat brain cell cultures. *J. Biol. Chem.* 274 35881–35888. 10.1074/jbc.274.50.35881 10585473

[B17] Bauer D. E. Jackson J. G. Genda E. N. Montoya M. M. Yudkoff M. Robinson M. B. (2012). The glutamate transporter, GLAST, participates in a macromolecular complex that supports glutamate metabolism. *Neurochem. Int.* 61 566–574. 10.1016/j.neuint.2012.01.013 22306776 PMC3350823

[B18] Bedner P. Niessen H. Odermatt B. Kretz M. Willecke K. Harz H. (2006). Selective permeability of different connexin channels to the second messenger cyclic AMP. *J. Biol. Chem.* 281 6673–6681. 10.1074/jbc.m511235200 16373337

[B19] Belanger M. Allaman I. Magistretti P. J. (2011). Brain energy metabolism: focus on astrocyte-neuron metabolic cooperation. *Cell Metab.* 14 724–738. 10.1016/j.cmet.2011.08.016 22152301

[B20] Ben Haim L. Carrillo-De Sauvage M. A. Ceyzeriat K. Escartin C. (2015). Elusive roles for reactive astrocytes in neurodegenerative diseases. *Front. Cell Neurosci.* 9:278. 10.3389/fncel.2015.00278 26283915 PMC4522610

[B21] Ben Haim L. Rowitch D. H. (2017). Functional diversity of astrocytes in neural circuit regulation. *Nat. Rev. Neurosci.* 18 31–41. 10.1038/nrn.2016.159 27904142

[B22] Bernard-Helary K. Ardourel M. Magistretti P. Hevor T. Cloix J. F. (2002). Stable transfection of cDNAs targeting specific steps of glycogen metabolism supports the existence of active gluconeogenesis in mouse cultured astrocytes. *Glia* 37 379–382. 10.1002/glia.10046 11870877

[B23] Bienert G. P. Moller A. L. Kristiansen K. A. Schulz A. Moller I. M. Schjoerring J. K. (2007). Specific aquaporins facilitate the diffusion of hydrogen peroxide across membranes. *J. Biol. Chem.* 282 1183–1192. 10.1074/jbc.m603761200 17105724

[B24] Bienert G. P. Schjoerring J. K. Jahn T. P. (2006). Membrane transport of hydrogen peroxide. *Biochim. Biophys. Acta Biomembr.* 1758 994–1003. 10.1016/j.bbamem.2006.02.015 16566894

[B25] Bird M. I. Munday L. A. Saggerson E. D. Clark J. B. (1985). Carnitine acyltransferase activities in rat brain mitochondria. Bimodal distribution, kinetic constants, regulation by malonyl-CoA and developmental pattern. *Biochem. J.* 226 323–330. 10.1042/bj2260323 3977877 PMC1144709

[B26] Blazquez C. Sanchez C. Velasco G. Guzman M. (1998). Role of carnitine palmitoyltransferase I in the control of ketogenesis in primary cultures of rat astrocytes. *J. Neurochem.* 71 1597–1606. 10.1046/j.1471-4159.1998.71041597.x 9751193

[B27] Blazquez C. Woods A. De Ceballos M. L. Carling D. Guzman M. (1999). The AMP-activated protein kinase is involved in the regulation of ketone body production by astrocytes. *J. Neurochem.* 73 1674–1682. 10.1046/j.1471-4159.1999.731674.x 10501215

[B28] Bolanos J. P. (2016). Bioenergetics and redox adaptations of astrocytes to neuronal activity. *J. Neurochem.* 139(Suppl. 2) 115–125. 10.1111/jnc.13486 26968531 PMC5018236

[B29] Booth R. F. Clark J. B. (1981). Energy metabolism in rat brain: inhibition of pyruvate decarboxylation by 3-hydroxybutyrate in neonatal mitochondria. *J. Neurochem.* 37 179–185. 10.1111/j.1471-4159.1981.tb05306.x 7252502

[B30] Boumezbeur F. Petersen K. F. Cline G. W. Mason G. F. Behar K. L. Shulman G. I. (2010). The contribution of blood lactate to brain energy metabolism in humans measured by dynamic 13C nuclear magnetic resonance spectroscopy. *J. Neurosci.* 30 13983–13991. 10.1523/jneurosci.2040-10.2010 20962220 PMC2996729

[B31] Bouzier-Sore A. K. Voisin P. Bouchaud V. Bezancon E. Franconi J. M. Pellerin L. (2006). Competition between glucose and lactate as oxidative energy substrates in both neurons and astrocytes: a comparative NMR study. *Eur. J. Neurosci.* 24 1687–1694. 10.1111/j.1460-9568.2006.05056.x 17004932

[B32] Brix B. Mesters J. R. Pellerin L. Johren O. (2012). Endothelial cell-derived nitric oxide enhances aerobic glycolysis in astrocytes via HIF-1alpha-mediated target gene activation. *J. Neurosci.* 32 9727–9735. 10.1523/jneurosci.0879-12.2012 22787058 PMC6622272

[B33] Cabodevilla A. G. Sanchez-Caballero L. Nintou E. Boiadjieva V. G. Picatoste F. Gubern A. (2013). Cell survival during complete nutrient deprivation depends on lipid droplet-fueled beta-oxidation of fatty acids. *J. Biol. Chem.* 288 27777–27788. 10.1074/jbc.m113.466656 23940052 PMC3784694

[B34] Cai S. Horne D. W. (2003). Transport of 5-formyltetrahydrofolate into primary cultured rat astrocytes. *Arch. Biochem. Biophys.* 410 161–166. 10.1016/s0003-9861(02)00663-x 12559989

[B35] Calabrese V. Ravagna A. Colombrita C. Scapagnini G. Guagliano E. Calvani M. (2005). Acetylcarnitine induces heme oxygenase in rat astrocytes and protects against oxidative stress: involvement of the transcription factor Nrf2. *J. Neurosci. Res.* 79 509–521. 10.1002/jnr.20386 15641110

[B36] Cardoso A. R. Chausse B. Da Cunha F. M. Luevano-Martinez L. A. Marazzi T. B. Pessoa P. S. (2012). Mitochondrial compartmentalization of redox processes. *Free Radic. Biol. Med.* 52 2201–2208. 10.1016/j.freeradbiomed.2012.03.008 22564526

[B37] Chechik T. Roeder L. M. Tildon J. T. Poduslo S. E. (1987). Ketone body enzyme activities in purified neurons, astrocytes and oligodendroglia. *Neurochem. Int.* 10 95–99. 10.1016/0197-0186(87)90179-3 20501089

[B38] Chen C. T. Trepanier M. O. Hopperton K. E. Domenichiello A. F. Masoodi M. Bazinet R. P. (2014). Inhibiting mitochondrial beta-oxidation selectively reduces levels of nonenzymatic oxidative polyunsaturated fatty acid metabolites in the brain. *J. Cereb. Blood Flow Metab.* 34 376–379. 10.1038/jcbfm.2013.221 24326387 PMC3948125

[B39] Chen Q. Vazquez E. J. Moghaddas S. Hoppel C. L. Lesnefsky E. J. (2003). Production of reactive oxygen species by mitochondria: central role of complex III. *J. Biol. Chem.* 278 36027–36031. 10.1074/jbc.m304854200 12840017

[B40] Chen Y. Vartiainen N. E. Ying W. Chan P. H. Koistinaho J. Swanson R. A. (2001). Astrocytes protect neurons from nitric oxide toxicity by a glutathione-dependent mechanism. *J. Neurochem.* 77 1601–1610. 10.1046/j.1471-4159.2001.00374.x 11413243

[B41] Choi I. Y. Tkac I. Ugurbil K. Gruetter R. (1999). Noninvasive measurements of [1-(13)C]glycogen concentrations and metabolism in rat brain in vivo. *J. Neurochem.* 73 1300–1308. 10.1046/j.1471-4159.1999.0731300.x 10461925

[B42] Chowdhury G. M. Jiang L. Rothman D. L. Behar K. L. (2014). The contribution of ketone bodies to basal and activity-dependent neuronal oxidation in vivo. *J. Cereb. Blood Flow Metab.* 34 1233–1242. 10.1038/jcbfm.2014.77 24780902 PMC4083391

[B43] Chung W. S. Allen N. J. Eroglu C. (2015). Astrocytes control synapse formation, function, and elimination. *Cold Spring Harb. Perspect. Biol.* 7:a020370. 10.1101/cshperspect.a020370 25663667 PMC4527946

[B44] Cocco T. Di Paola M. Papa S. Lorusso M. (1999). Arachidonic acid interaction with the mitochondrial electron transport chain promotes reactive oxygen species generation. *Free Radic. Biol. Med.* 27 51–59. 10.1016/s0891-5849(99)00034-9 10443919

[B45] Courchesne-Loyer A. Croteau E. Castellano C. A. St-Pierre V. Hennebelle M. Cunnane S. C. (2017). Inverse relationship between brain glucose and ketone metabolism in adults during short-term moderate dietary ketosis: A dual tracer quantitative positron emission tomography study. *J. Cereb. Blood Flow Metab.* 37 2485–2493. 10.1177/0271678x16669366 27629100 PMC5531346

[B46] Cullingford T. E. Dolphin C. T. Bhakoo K. K. Peuchen S. Canevari L. Clark J. B. (1998). Molecular cloning of rat mitochondrial 3-hydroxy-3-methylglutaryl-CoA lyase and detection of the corresponding mRNA and of those encoding the remaining enzymes comprising the ketogenic 3-hydroxy-3-methylglutaryl-CoA cycle in central nervous system of suckling rat. *Biochem. J.* 329(Pt 2) 373–381. 10.1042/bj3290373 9425122 PMC1219054

[B47] Cullingford T. E. Dolphin C. T. Sato H. (2002). The peroxisome proliferator-activated receptor alpha-selective activator ciprofibrate upregulates expression of genes encoding fatty acid oxidation and ketogenesis enzymes in rat brain. *Neuropharmacology* 42 724–730. 10.1016/s0028-3908(02)00014-x 11985831

[B48] Delgado-Esteban M. Almeida A. Bolanos J. P. (2000). D-Glucose prevents glutathione oxidation and mitochondrial damage after glutamate receptor stimulation in rat cortical primary neurons. *J. Neurochem.* 75 1618–1624. 10.1046/j.1471-4159.2000.0751618.x 10987843

[B49] Diaz-Garcia C. M. Mongeon R. Lahmann C. Koveal D. Zucker H. Yellen G. (2017). Neuronal stimulation triggers neuronal Glycolysis and not lactate uptake. *Cell Metab* 26 361–374.e364. 28768175 10.1016/j.cmet.2017.06.021 PMC5559896

[B50] Dringen R. Gutterer J. M. Gros C. Hirrlinger J. (2001). Aminopeptidase N mediates the utilization of the GSH precursor CysGly by cultured neurons. *J. Neurosci. Res.* 66 1003–1008. 10.1002/jnr.10042 11746430

[B51] Dringen R. Kranich O. Hamprecht B. (1997). The gamma-glutamyl transpeptidase inhibitor acivicin preserves glutathione released by astroglial cells in culture. *Neurochem. Res.* 22 727–733. 9178957 10.1023/a:1027310328310

[B52] Dringen R. Pfeiffer B. Hamprecht B. (1999). Synthesis of the antioxidant glutathione in neurons: supply by astrocytes of CysGly as precursor for neuronal glutathione. *J. Neurosci.* 19 562–569. 10.1523/jneurosci.19-02-00562.1999 9880576 PMC6782200

[B53] Drulis-Fajdasz D. Gizak A. Wojtowicz T. Wisniewski J. R. Rakus D. (2018). Aging-associated changes in hippocampal glycogen metabolism in mice. Evidence for and against astrocyte-to-neuron lactate shuttle. *Glia* 66 1481–1495. 10.1002/glia.23319 29493012 PMC6001795

[B54] Ducker G. S. Chen L. Morscher R. J. Ghergurovich J. M. Esposito M. Teng X. (2016). Reversal of cytosolic one-carbon flux compensates for loss of the mitochondrial folate pathway. *Cell Metab.* 24 640–641. 10.1016/j.cmet.2016.09.011 27732838

[B55] Ebert D. Haller R. G. Walton M. E. (2003). Energy contribution of octanoate to intact rat brain metabolism measured by 13C nuclear magnetic resonance spectroscopy. *J. Neurosci.* 23 5928–5935. 10.1523/jneurosci.23-13-05928.2003 12843297 PMC6741266

[B56] Edmond J. Robbins R. A. Bergstrom J. D. Cole R. A. De Vellis J. (1987). Capacity for substrate utilization in oxidative metabolism by neurons, astrocytes, and oligodendrocytes from developing brain in primary culture. *J. Neurosci. Res.* 18 551–561. 10.1002/jnr.490180407 3481403

[B57] Eraso-Pichot A. Braso-Vives M. Golbano A. Menacho C. Claro E. Galea E. (2018). GSEA of mouse and human mitochondriomes reveals fatty acid oxidation in astrocytes. *Glia* 66 1724–1735. 10.1002/glia.23330 29575211

[B58] Escartin C. Pierre K. Colin A. Brouillet E. Delzescaux T. Guillermier M. (2007). Activation of astrocytes by CNTF induces metabolic plasticity and increases resistance to metabolic insults. *J. Neurosci.* 27 7094–7104. 10.1523/jneurosci.0174-07.2007 17611262 PMC6794576

[B59] Fan J. Ye J. Kamphorst J. J. Shlomi T. Thompson C. B. Rabinowitz J. D. (2014). Quantitative flux analysis reveals folate-dependent NADPH production. *Nature* 510 298–302. 10.1038/nature13236 24805240 PMC4104482

[B60] Farmer B. C. Kluemper J. Johnson L. A. (2019). Apolipoprotein E4 alters astrocyte fatty acid metabolism and lipid droplet formation. *Cells* 8:182. 10.3390/cells8020182 30791549 PMC6406677

[B61] Farmer W. T. Abrahamsson T. Chierzi S. Lui C. Zaelzer C. Jones E. V. (2016). Neurons diversify astrocytes in the adult brain through sonic hedgehog signaling. *Science* 351 849–854. 10.1126/science.aab3103 26912893

[B62] Fecher C. Trovo L. Muller S. A. Snaidero N. Wettmarshausen J. Heink S. (2019). Cell-type-specific profiling of brain mitochondria reveals functional and molecular diversity. *Nat. Neurosci.* 22 1731–1742. 10.1038/s41593-019-0479-z 31501572

[B63] Fedorovich S. V. Voronina P. P. Waseem T. V. (2018). Ketogenic diet versus ketoacidosis: what determines the influence of ketone bodies on neurons? *Neural Regen. Res.* 13 2060–2063. 10.4103/1673-5374.241442 30323121 PMC6199956

[B64] Fitzner D. Bader J. M. Penkert H. Bergner C. G. Su M. Weil M. T. (2020). Cell-Type- and brain-region-resolved mouse brain lipidome. *Cell Rep.* 32:108132. 10.1016/j.celrep.2020.108132 32937123

[B65] Foo L. C. Dougherty J. D. (2013). Aldh1L1 is expressed by postnatal neural stem cells in vivo. *Glia* 61 1533–1541. 10.1002/glia.22539 23836537 PMC3777382

[B66] Foster D. W. (2012). Malonyl-CoA: the regulator of fatty acid synthesis and oxidation. *J. Clin. Invest.* 122 1958–1959. 10.1172/jci63967 22833869 PMC3366419

[B67] Garber A. J. Ballard F. J. (1969). Phosphoenolpyruvate synthesis and release by mitochondria from guinea pig liver. *J. Biol. Chem.* 244 4696–4703. 5808512

[B68] Garcia-Marin V. Garcia-Lopez P. Freire M. (2007). Cajal’s contributions to glia research. *Trends Neurosci.* 30 479–487. 10.1016/j.tins.2007.06.008 17765327

[B69] Garcia-Nogales P. Almeida A. Bolanos J. P. (2003). Peroxynitrite protects neurons against nitric oxide-mediated apoptosis. A key role for glucose-6-phosphate dehydrogenase activity in neuroprotection. *J. Biol. Chem.* 278 864–874. 10.1074/jbc.m206835200 12414804

[B70] Genda E. N. Jackson J. G. Sheldon A. L. Locke S. F. Greco T. M. O’donnell J. C. (2011). Co-compartmentalization of the astroglial glutamate transporter, GLT-1, with glycolytic enzymes and mitochondria. *J. Neurosci.* 31 18275–18288. 10.1523/jneurosci.3305-11.2011 22171032 PMC3259858

[B71] Ghosh A. Cheung Y. Y. Mansfield B. C. Chou J. Y. (2005). Brain contains a functional glucose-6-phosphatase complex capable of endogenous glucose production. *J. Biol. Chem.* 280 11114–11119. 10.1074/jbc.m410894200 15661744

[B72] Gnaedinger J. M. Miller J. C. Latker C. H. Rapoport S. I. (1988). Cerebral metabolism of plasma [^14^C] palmitate in awake, adult rat: subcellular localization. *Neurochem. Res.* 13 21–29. 10.1007/BF00971850 3368026

[B73] Goldberg G. S. Moreno A. P. Lampe P. D. (2002). Gap junctions between cells expressing connexin 43 or 32 show inverse permselectivity to adenosine and ATP. *J. Biol. Chem.* 277 36725–36730. 10.1074/jbc.m109797200 12119284

[B74] Goncalves C. A. Rodrigues L. Bobermin L. D. Zanotto C. Vizuete A. Quincozes-Santos A. (2018). Glycolysis-derived compounds from astrocytes that modulate synaptic communication. *Front. Neurosci.* 12:1035 10.3389/fncel.2015.001035 PMC6351787 30728759

[B75] Gonzalez-Gutierrez A. Ibacache A. Esparza A. Barros L. F. Sierralta J. (2019). Neuronal lactate levels depend on glia-derived lactate during high brain activity in *Drosophila*. *Glia* 68 1213–1227. 10.1002/glia.23772 31876077

[B76] Goubert E. Mircheva Y. Lasorsa F. M. Melon C. Profilo E. Sutera J. (2017). Inhibition of the mitochondrial glutamate carrier SLC25A22 in astrocytes leads to intracellular glutamate accumulation. *Front. Cell Neurosci.* 11:149. 10.3389/fncel.2015.00149 28620281 PMC5449474

[B77] Grivennikova V. G. Vinogradov A. D. (2006). Generation of superoxide by the mitochondrial Complex I. *Biochim. Biophys. Acta* 1757 553–561. 10.1016/j.bbabio.2006.03.013 16678117

[B78] Guzman M. Blazquez C. (2001). Is there an astrocyte-neuron ketone body shuttle? *Trends Endocrinol. Metab.* 12 169–173. 10.1016/s1043-2760(00)00370-2 11295573

[B79] Gzielo K. Soltys Z. Rajfur Z. Setkowicz Z. K. (2019). The Impact of the ketogenic diet on glial cells morphology. A quantitative morphological analysis. *Neuroscience* 413 239–251. 10.1016/j.neuroscience.2019.06.009 31220541

[B80] Halestrap A. P. (1978). Pyruvate and ketone-body transport across the mitochondrial membrane. Exchange properties, pH-dependence and mechanism of the carrier. *Biochem. J.* 172 377–387. 10.1042/bj1720377 28726 PMC1185711

[B81] Halestrap A. P. Price N. T. (1999). The proton-linked monocarboxylate transporter (MCT) family: structure, function and regulation. *Biochem. J.* 343(Pt 2) 281–299. 10.1042/0264-6021:3430281 10510291 PMC1220552

[B82] Halim N. D. Mcfate T. Mohyeldin A. Okagaki P. Korotchkina L. G. Patel M. S. (2010). Phosphorylation status of pyruvate dehydrogenase distinguishes metabolic phenotypes of cultured rat brain astrocytes and neurons. *Glia* 58 1168–1176. 10.1002/glia.20996 20544852 PMC2915787

[B83] Hamilton J. A. Brunaldi K. (2007). A model for fatty acid transport into the brain. *J. Mol. Neurosci.* 33 12–17. 10.1007/s12031-007-0050-3 17901540

[B84] Han D. Antunes F. Canali R. Rettori D. Cadenas E. (2003). Voltage-dependent anion channels control the release of the superoxide anion from mitochondria to cytosol. *J. Biol. Chem.* 278 5557–5563. 10.1074/jbc.m210269200 12482755

[B85] Hein S. Schonfeld P. Kahlert S. Reiser G. (2008). Toxic effects of X-linked adrenoleukodystrophy-associated, very long chain fatty acids on glial cells and neurons from rat hippocampus in culture. *Hum. Mol. Genet.* 17 1750–1761. 10.1093/hmg/ddn066 18344355

[B86] Herrero-Mendez A. Almeida A. Fernandez E. Maestre C. Moncada S. Bolanos J. P. (2009). The bioenergetic and antioxidant status of neurons is controlled by continuous degradation of a key glycolytic enzyme by APC/C-Cdh1. *Nat. Cell Biol.* 11 747–752. 10.1038/ncb1881 19448625

[B87] Hertz L. (2011). Brain glutamine synthesis requires neuronal aspartate: a commentary. *J. Cereb. Blood Flow Metab.* 31 384–387. 10.1038/jcbfm.2010.199 21063427 PMC3049474

[B88] Hirrlinger J. Dringen R. (2005). Multidrug resistance protein 1-mediated export of glutathione and glutathione disulfide from brain astrocytes. *Methods Enzymol.* 400 395–409. 10.1016/s0076-6879(05)00023-6 16399362

[B89] Hirrlinger J. Schulz J. B. Dringen R. (2002). Glutathione release from cultured brain cells: multidrug resistance protein 1 mediates the release of GSH from rat astroglial cells. *J. Neurosci. Res.* 69 318–326. 10.1002/jnr.10308 12125073

[B90] Ho J. K. Moser H. Kishimoto Y. Hamilton J. A. (1995). Interactions of a very long chain fatty acid with model membranes and serum albumin. Implications for the pathogenesis of adrenoleukodystrophy. *J. Clin. Invest.* 96 1455–1463. 10.1172/jci118182 7657817 PMC185769

[B91] Hofmann K. Rodriguez-Rodriguez R. Gaebler A. Casals N. Scheller A. Kuerschner L. (2017). Astrocytes and oligodendrocytes in grey and white matter regions of the brain metabolize fatty acids. *Sci. Rep.* 7:10779. 10.1038/s41598-017-11103-5 PMC5589817 28883484

[B92] Houten S. M. Wanders R. J. (2010). A general introduction to the biochemistry of mitochondrial fatty acid beta-oxidation. *J. Inherit. Metab. Dis.* 33 469–477. 10.1007/s10545-010-9061-2 20195903 PMC2950079

[B93] Hovik R. Brodal B. Bartlett K. Osmundsen H. (1991). Metabolism of acetyl-CoA by isolated peroxisomal fractions: formation of acetate and acetoacetyl-CoA. *J. Lipid Res.* 32 993–999. 1682408

[B94] Hyder F. Rothman D. L. Bennett M. R. (2013). Cortical energy demands of signaling and nonsignaling components in brain are conserved across mammalian species and activity levels. *Proc. Natl. Acad. Sci. U.S.A.* 110 3549–3554. 10.1073/pnas.1214912110 23319606 PMC3587194

[B95] Ioannou M. S. Jackson J. Sheu S. H. Chang C. L. Weigel A. V. Liu H. (2019). Neuron-astrocyte metabolic coupling protects against activity-induced fatty acid toxicity. *Cell* 177 1522–1535.e1514. 31130380 10.1016/j.cell.2019.04.001

[B96] Jackson J. G. O’donnell J. C. Takano H. Coulter D. A. Robinson M. B. (2014). Neuronal activity and glutamate uptake decrease mitochondrial mobility in astrocytes and position mitochondria near glutamate transporters. *J. Neurosci.* 34 1613–1624. 10.1523/jneurosci.3510-13.2014 24478345 PMC3905137

[B97] Jackson J. G. Robinson M. B. (2018). Regulation of mitochondrial dynamics in astrocytes: mechanisms, consequences, and unknowns. *Glia* 66 1213–1234. 10.1002/glia.23252 29098734 PMC5904024

[B98] Jakoby P. Schmidt E. Ruminot I. Gutierrez R. Barros L. F. Deitmer J. W. (2014). Higher transport and metabolism of glucose in astrocytes compared with neurons: a multiphoton study of hippocampal and cerebellar tissue slices. *Cereb. Cortex* 24 222–231. 10.1093/cercor/bhs309 23042735

[B99] Jansen G. A. Wanders R. J. (2006). Alpha-oxidation. *Biochim. Biophys. Acta* 1763 1403–1412. 16934890 10.1016/j.bbamcr.2006.07.012

[B100] Jernberg J. N. Bowman C. E. Wolfgang M. J. Scafidi S. (2017). Developmental regulation and localization of carnitine palmitoyltransferases (CPTs) in rat brain. *J. Neurochem.* 142 407–419. 10.1111/jnc.14072 28512781 PMC5927624

[B101] Jimenez-Blasco D. Santofimia-Castano P. Gonzalez A. Almeida A. Bolanos J. P. (2015). Astrocyte NMDA receptors’ activity sustains neuronal survival through a Cdk5-Nrf2 pathway. *Cell Death. Differ.* 22 1877–1889. 10.1038/cdd.2015.49 25909891 PMC4648333

[B102] Jones L. L. Mcdonald D. A. Borum P. R. (2010). Acylcarnitines: role in brain. *Prog. Lipid Res.* 49 61–75. 10.1016/j.plipres.2009.08.004 19720082

[B103] Kempson S. A. Zhou Y. Danbolt N. C. (2014). The betaine/GABA transporter and betaine: roles in brain, kidney, and liver. *Front. Physiol.* 5:159. 10.3389/fncel.2015.00159 24795654 PMC4006062

[B104] Kimura S. Amemiya F. (1990). Brain and liver pathology in a patient with carnitine deficiency. *Brain Dev.* 12 436–439. 10.1016/s0387-7604(12)80079-9 2240466

[B105] Komen J. C. Distelmaier F. Koopman W. J. Wanders R. J. Smeitink J. Willems P. H. (2007). Phytanic acid impairs mitochondrial respiration through protonophoric action. *Cell Mol. Life. Sci.* 64 3271–3281. 10.1007/s00018-007-7357-7 17968498 PMC11135980

[B106] Konttinen H. Gureviciene I. Oksanen M. Grubman A. Loppi S. Huuskonen M. T. (2019). PPARbeta/delta-agonist GW0742 ameliorates dysfunction in fatty acid oxidation in PSEN1DeltaE9 astrocytes. *Glia* 67 146–159. 10.1002/glia.23534 30453390 PMC7526864

[B107] Korshunov S. S. Korkina O. V. Ruuge E. K. Skulachev V. P. Starkov A. A. (1998). Fatty acids as natural uncouplers preventing generation of O_2_.- and H_2_O_2_ by mitochondria in the resting state. *FEBS Lett.* 435 215–218. 10.1016/s0014-5793(98)01073-4 9762912

[B108] Kory N. Wyant G. A. Prakash G. Uit De Bos J. Bottanelli F. Pacold M. E. (2018). SFXN1 is a mitochondrial serine transporter required for one-carbon metabolism. *Science* 362:eaat9528. 10.1126/science.aat9528 30442778 PMC6300058

[B109] Koulakoff A. Ezan P. Giaume C. (2008). Neurons control the expression of connexin 30 and connexin 43 in mouse cortical astrocytes. *Glia* 56 1299–1311. 10.1002/glia.20698 18512249

[B110] Kranich O. Hamprecht B. Dringen R. (1996). Different preferences in the utilization of amino acids for glutathione synthesis in cultured neurons and astroglial cells derived from rat brain. *Neurosci. Lett.* 219 211–214. 10.1016/s0304-3940(96)13217-1 8971817

[B111] Krencik R. Weick J. P. Liu Y. Zhang Z. J. Zhang S. C. (2011). Specification of transplantable astroglial subtypes from human pluripotent stem cells. *Nat. Biotechnol.* 29 528–534. 10.1038/nbt.1877 21602806 PMC3111840

[B112] Kruska N. Schonfeld P. Pujol A. Reiser G. (2015). Astrocytes and mitochondria from adrenoleukodystrophy protein (ABCD1)-deficient mice reveal that the adrenoleukodystrophy-associated very long-chain fatty acids target several cellular energy-dependent functions. *Biochim. Biophys. Acta* 1852 925–936. 10.1016/j.bbadis.2015.01.005 25583114

[B113] Kunnecke B. Cerdan S. Seelig J. (1993). Cerebral metabolism of [1,2-13C2]glucose and [U-13C4]3-hydroxybutyrate in rat brain as detected by 13C NMR spectroscopy. *NMR Biomed.* 6 264–277. 10.1002/nbm.1940060406 8105858

[B114] Lapato A. S. Tiwari-Woodruff S. K. (2018). Connexins and pannexins: at the junction of neuro-glial homeostasis & disease. *J. Neurosci. Res.* 96 31–44. 10.1002/jnr.24088 28580666 PMC5749981

[B115] Lavrentyev E. N. Matta S. G. Cook G. A. (2004). Expression of three carnitine palmitoyltransferase-I isoforms in 10 regions of the rat brain during feeding, fasting, and diabetes. *Biochem. Biophys. Res. Commun.* 315 174–178. 10.1016/j.bbrc.2004.01.040 15013442

[B116] Lawrence S. A. Hackett J. C. Moran R. G. (2011). Tetrahydrofolate recognition by the mitochondrial folate transporter. *J. Biol. Chem.* 286 31480–31489. 10.1074/jbc.m111.272187 21768094 PMC3173085

[B117] Le Foll C. Levin B. E. (2016). Fatty acid-induced astrocyte ketone production and the control of food intake. *Am. J. Physiol. Regul. Integr. Comp. Physiol.* 310 R1186–R1192. 27122369 10.1152/ajpregu.00113.2016 PMC4935491

[B118] Lee J. Wolfgang M. J. (2012). Metabolomic profiling reveals a role for CPT1c in neuronal oxidative metabolism. *BMC Biochem.* 13:23. 10.1186/1471-2091-13-23 23098614 PMC3523003

[B119] Leke R. Schousboe A. (2016). The glutamine transporters and their role in the glutamate/GABA-glutamine cycle. *Adv. Neurobiol.* 13 223–257. 10.1007/978-3-319-45096-4_8 27885631

[B120] Leung K. Y. Pai Y. J. Chen Q. Santos C. Calvani E. Sudiwala S. (2017). Partitioning of one-carbon units in folate and methionine metabolism is essential for neural tube closure. *Cell Rep.* 21 1795–1808. 10.1016/j.celrep.2017.10.072 29141214 PMC5699646

[B121] Lewis C. A. Parker S. J. Fiske B. P. Mccloskey D. Gui D. Y. Green C. R. (2014). Tracing compartmentalized NADPH metabolism in the cytosol and mitochondria of mammalian cells. *Mol. Cell.* 55 253–263. 10.1016/j.molcel.2014.05.008 24882210 PMC4106038

[B122] Li S. Uno Y. Rudolph U. Cobb J. Liu J. Anderson T. (2018). Astrocytes in primary cultures express serine racemase, synthesize d-serine and acquire A1 reactive astrocyte features. *Biochem. Pharmacol.* 151 245–251. 10.1016/j.bcp.2017.12.023 29305854 PMC5899945

[B123] Lin H. Patel S. Affleck V. S. Wilson I. Turnbull D. M. Joshi A. R. (2017). Fatty acid oxidation is required for the respiration and proliferation of malignant glioma cells. *Neurol. Oncol.* 19 43–54. 10.1093/neuonc/now128 27365097 PMC5193020

[B124] Liu L. Mackenzie K. R. Putluri N. Maletic-Savatic M. Bellen H. J. (2017). The glia-neuron lactate shuttle and elevated ROS promote lipid synthesis in neurons and lipid droplet accumulation in glia via APOE/D. *Cell Metab.* 26 719–737.e716. 28965825 10.1016/j.cmet.2017.08.024 PMC5677551

[B125] Liu L. Zhang K. Sandoval H. Yamamoto S. Jaiswal M. Sanz E. (2015). Glial lipid droplets and ROS induced by mitochondrial defects promote neurodegeneration. *Cell* 160 177–190. 10.1016/j.cell.2014.12.019 25594180 PMC4377295

[B126] Lopert P. Patel M. (2014). Nicotinamide nucleotide transhydrogenase (Nnt) links the substrate requirement in brain mitochondria for hydrogen peroxide removal to the thioredoxin/peroxiredoxin (Trx/Prx) system. *J. Biol. Chem.* 289 15611–15620. 10.1074/jbc.m113.533653 24722990 PMC4140916

[B127] Lopes-Cardozo M. Larsson O. M. Schousboe A. (1986). Acetoacetate and glucose as lipid precursors and energy substrates in primary cultures of astrocytes and neurons from mouse cerebral cortex. *J. Neurochem.* 46 773–778. 10.1111/j.1471-4159.1986.tb13039.x 3081684

[B128] Lopez-Fabuel I. Le Douce J. Logan A. James A. M. Bonvento G. Murphy M. P. (2016). Complex I assembly into supercomplexes determines differential mitochondrial ROS production in neurons and astrocytes. *Proc. Natl. Acad. Sci. U.S.A.* 113 13063–13068. 10.1073/pnas.1613701113 27799543 PMC5135366

[B129] Lovatt D. Sonnewald U. Waagepetersen H. S. Schousboe A. He W. Lin J. H. (2007). The transcriptome and metabolic gene signature of protoplasmic astrocytes in the adult murine cortex. *J. Neurosci.* 27 12255–12266. 10.1523/jneurosci.3404-07.2007 17989291 PMC6673251

[B130] Lundgaard I. Li B. Xie L. Kang H. Sanggaard S. Haswell J. D. (2015). Direct neuronal glucose uptake heralds activity-dependent increases in cerebral metabolism. *Nat. Commun.* 6:6807. 10.1038/ncomms7807 PMC4410436 25904018

[B131] Ma Y. Wang W. Devarakonda T. Zhou H. Wang X. Y. Salloum F. N. (2020). Functional analysis of molecular and pharmacological modulators of mitochondrial fatty acid oxidation. *Sci. Rep.* 10:1450. 10.1038/s41598-020-58334-7 PMC6989517 31996743

[B132] Machler P. Wyss M. T. Elsayed M. Stobart J. Gutierrez R. Von Faber-Castell A. (2016). In *Vivo* evidence for a lactate gradient from astrocytes to neurons. *Cell Metab.* 23 94–102. 10.1016/j.cmet.2015.10.010 26698914

[B133] MacVicar B. A. Newman E. A. (2015). Astrocyte regulation of blood flow in the brain. *Cold Spring Harb. Perspect. Biol.* 7:a020388. 10.1101/cshperspect.a020388 25818565 PMC4448617

[B134] Magistretti P. J. Allaman I. (2015). A cellular perspective on brain energy metabolism and functional imaging. *Neuron* 86 883–901. 10.1016/j.neuron.2015.03.035 25996133

[B135] Magistretti P. J. Pellerin L. Rothman D. L. Shulman R. G. (1999). Energy on demand. *Science* 283 496–497. 9988650 10.1126/science.283.5401.496

[B136] Marszalek J. R. Kitidis C. Dararutana A. Lodish H. F. (2004). Acyl-CoA synthetase 2 overexpression enhances fatty acid internalization and neurite outgrowth. *J. Biol. Chem.* 279 23882–23891. 10.1074/jbc.m313460200 15051725

[B137] Mashek D. G. Li L. O. Coleman R. A. (2007). Long-chain acyl-CoA synthetases and fatty acid channeling. *Future Lipidol.* 2 465–476. 10.2217/17460875.2.4.465 20354580 PMC2846691

[B138] Matthews G. D. Gur N. Koopman W. J. Pines O. Vardimon L. (2010). Weak mitochondrial targeting sequence determines tissue-specific subcellular localization of glutamine synthetase in liver and brain cells. *J. Cell Sci.* 123 351–359. 10.1242/jcs.060749 20053634

[B139] McKenna M. C. Sonnewald U. Huang X. Stevenson J. Zielke H. R. (1996). Exogenous glutamate concentration regulates the metabolic fate of glutamate in astrocytes. *J. Neurochem.* 66 386–393. 10.1046/j.1471-4159.1996.66010386.x 8522979

[B140] McKenna M. C. Stridh M. H. Mcnair L. F. Sonnewald U. Waagepetersen H. S. Schousboe A. (2016). Glutamate oxidation in astrocytes: roles of glutamate dehydrogenase and aminotransferases. *J. Neurosci. Res.* 94 1561–1571. 10.1002/jnr.23908 27629247

[B141] McKenna M. C. Tildon J. T. Stevenson J. H. Huang X. Kingwell K. G. (1995). Regulation of mitochondrial and cytosolic malic enzymes from cultured rat brain astrocytes. *Neurochem. Res.* 20 1491–1501. 10.1007/bf00970599 8789613

[B142] Minich T. Yokota S. Dringen R. (2003). Cytosolic and mitochondrial isoforms of NADP+-dependent isocitrate dehydrogenases are expressed in cultured rat neurons, astrocytes, oligodendrocytes and microglial cells. *J. Neurochem.* 86 605–614. 10.1046/j.1471-4159.2003.01871.x 12859674

[B143] Mitchell R. W. On N. H. Del Bigio M. R. Miller D. W. Hatch G. M. (2011). Fatty acid transport protein expression in human brain and potential role in fatty acid transport across human brain microvessel endothelial cells. *J. Neurochem.* 117 735–746. 10.1111/j.1471-4159.2011.07245.x 21395585

[B144] Morris A. A. (2005). Cerebral ketone body metabolism. *J. Inherit. Metab. Dis.* 28 109–121. 15877199 10.1007/s10545-005-5518-0

[B145] Morris G. Maes M. Berk M. Carvalho A. F. Puri B. K. (2020). Nutritional ketosis as an intervention to relieve astrogliosis: possible therapeutic applications in the treatment of neurodegenerative and neuroprogressive disorders. *Eur. Psychiatry* 63:e8. 10.1192/j.eurpsy.2019.13 PMC8057392 32093791

[B146] Muller M. S. Fouyssac M. Taylor C. W. (2018). Effective glucose uptake by human astrocytes requires its sequestration in the endoplasmic reticulum by Glucose-6-phosphatase-beta. *Curr. Biol.* 28 3481–3486.e3484. 30415704 10.1016/j.cub.2018.08.060 PMC6224479

[B147] Murin R. Cesar M. Kowtharapu B. S. Verleysdonk S. Hamprecht B. (2009). Expression of pyruvate carboxylase in cultured oligodendroglial, microglial and ependymal cells. *Neurochem. Res.* 34 480–489. 10.1007/s11064-008-9806-6 18686030

[B148] Neymeyer V. Tephly T. R. Miller M. W. (1997). Folate and 10-formyltetrahydrofolate dehydrogenase (FDH) expression in the central nervous system of the mature rat. *Brain Res.* 766 195–204. 10.1016/s0006-8993(97)00528-3 9359603

[B149] Niessen H. Harz H. Bedner P. Kramer K. Willecke K. (2000). Selective permeability of different connexin channels to the second messenger inositol 1,4,5-trisphosphate. *J. Cell Sci.* 113(Pt 8) 1365–1372. 10725220 10.1242/jcs.113.8.1365

[B150] Nissen J. D. Lykke K. Bryk J. Stridh M. H. Zaganas I. Skytt D. M. (2017). Expression of the human isoform of glutamate dehydrogenase, hGDH2, augments TCA cycle capacity and oxidative metabolism of glutamate during glucose deprivation in astrocytes. *Glia* 65 474–488. 10.1002/glia.23105 28032919

[B151] Nissen J. D. Pajecka K. Stridh M. H. Skytt D. M. Waagepetersen H. S. (2015). Dysfunctional TCA-cycle metabolism in glutamate Dehydrogenase deficient astrocytes. *Glia* 63 2313–2326. 10.1002/glia.22895 26221781

[B152] Norenberg M. D. Martinez-Hernandez A. (1979). Fine structural localization of glutamine synthetase in astrocytes of rat brain. *Brain Res.* 161 303–310. 10.1016/0006-8993(79)90071-4 31966

[B153] Obici S. Feng Z. Arduini A. Conti R. Rossetti L. (2003). Inhibition of hypothalamic carnitine palmitoyltransferase-1 decreases food intake and glucose production. *Nat. Med.* 9 756–761. 10.1038/nm873 12754501

[B154] Ohnishi T. Balan S. Toyoshima M. Maekawa M. Ohba H. Watanabe A. (2019). Investigation of betaine as a novel psychotherapeutic for schizophrenia. *eBio Med.* 45 432–446. 10.1016/j.ebiom.2019.05.062 31255657 PMC6642071

[B155] Olivier L. M. Kovacs W. Masuda K. Keller G. A. Krisans S. K. (2000). Identification of peroxisomal targeting signals in cholesterol biosynthetic enzymes. AA-CoA thiolase, hmg-coa synthase, MPPD, and FPP synthase. *J. Lipid Res.* 41 1921–1935. 11108725

[B156] Olsen G. M. Sonnewald U. (2015). Glutamate: where does it come from and where does it go? *Neurochem. Int.* 88 47–52. 10.1016/j.neuint.2014.11.006 25447768

[B157] Orthmann-Murphy J. L. Abrams C. K. Scherer S. S. (2008). Gap junctions couple astrocytes and oligodendrocytes. *J. Mol. Neurosci.* 35 101–116. 10.1007/s12031-007-9027-5 18236012 PMC2650399

[B158] Ouellet M. Emond V. Chen C. T. Julien C. Bourasset F. Oddo S. (2009). Diffusion of docosahexaenoic and eicosapentaenoic acids through the blood-brain barrier: an *in situ* cerebral perfusion study. *Neurochem. Int.* 55 476–482. 10.1016/j.neuint.2009.04.018 19442696

[B159] Owen O. E. Kalhan S. C. Hanson R. W. (2002). The key role of anaplerosis and cataplerosis for citric acid cycle function. *J. Biol. Chem.* 277 30409–30412. 10.1074/jbc.r200006200 12087111

[B160] Owen O. E. Morgan A. P. Kemp H. G. Sullivan J. M. Herrera M. G. (1967). Brain metabolism during fasting. *J. Clin. Invest.* 46 1589–1595. 6061736 10.1172/JCI105650 PMC292907

[B161] Pai Y. J. Leung K. Y. Savery D. Hutchin T. Prunty H. Heales S. (2015). Glycine decarboxylase deficiency causes neural tube defects and features of non-ketotic hyperglycinemia in mice. *Nat. Commun.* 6:6388. 10.1038/ncomms7388 PMC4366506 25736695

[B162] Pajecka K. Nissen J. D. Stridh M. H. Skytt D. M. Schousboe A. Waagepetersen H. S. (2015). Glucose replaces glutamate as energy substrate to fuel glutamate uptake in glutamate dehydrogenase-deficient astrocytes. *J. Neurosci. Res.* 93 1093–1100. 10.1002/jnr.23568 25656783

[B163] Panov A. Orynbayeva Z. Vavilin V. Lyakhovich V. (2014). Fatty acids in energy metabolism of the central nervous system. *Biomed. Res. Int.* 2014:472459. 10.1155/2014/472459 PMC4026875 24883315

[B164] Paradies G. Papa S. (1977). On the kinetics and substrate specificity of the pyruvate translocator in rat liver mitochondria. *Biochim. Biophys. Acta* 462 333–346. 10.1016/0005-2728(77)90132-3 588572

[B165] Patel A. B. Lai J. C. Chowdhury G. M. Hyder F. Rothman D. L. Shulman R. G. (2014). Direct evidence for activity-dependent glucose phosphorylation in neurons with implications for the astrocyte-to-neuron lactate shuttle. *Proc. Natl. Acad. Sci. U.S.A.* 111 5385–5390. 10.1073/pnas.1403576111 24706914 PMC3986127

[B166] Pellerin L. Magistretti P. J. (1994). Glutamate uptake into astrocytes stimulates aerobic glycolysis: a mechanism coupling neuronal activity to glucose utilization. *Proc. Natl. Acad. Sci. U.S.A.* 91 10625–10629. 10.1073/pnas.91.22.10625 7938003 PMC45074

[B167] Polyzos A. A. Lee D. Y. Datta R. Hauser M. Budworth H. Holt A. (2019). Metabolic reprogramming in astrocytes distinguishes region-specific neuronal susceptibility in huntington mice. *Cell Metab.* 29 1258–1273.e1211. 30930170 10.1016/j.cmet.2019.03.004 PMC6583797

[B168] Purdon D. Arai T. Rapoport S. (1997). No evidence for direct incorporation of esterified palmitic acid from plasma into brain lipids of awake adult rat. *J. Lipid Res.* 38 526–530. 9101433

[B169] Quinlan C. L. Perevoshchikova I. V. Hey-Mogensen M. Orr A. L. Brand M. D. (2013). Sites of reactive oxygen species generation by mitochondria oxidizing different substrates. *Redox Biol.* 1 304–312. 10.1016/j.redox.2013.04.005 24024165 PMC3757699

[B170] Riveros N. Fiedler J. Lagos N. Munoz C. Orrego F. (1986). Glutamate in rat brain cortex synaptic vesicles: influence of the vesicle isolation procedure. *Brain Res.* 386 405–408. 10.1016/0006-8993(86)90181-2 2877717

[B171] Rolfe D. F. Brown G. C. (1997). Cellular energy utilization and molecular origin of standard metabolic rate in mammals. *Physiol. Rev.* 77 731–758. 10.1152/physrev.1997.77.3.731 9234964

[B172] Romano A. Koczwara J. B. Gallelli C. A. Vergara D. Micioni Di Bonaventura M. V. Gaetani S. (2017). Fats for thoughts: an update on brain fatty acid metabolism. *Int. J. Biochem. Cell Biol.* 84 40–45. 10.1016/j.biocel.2016.12.015 28065757

[B173] Rosafio K. Pellerin L. (2014). Oxygen tension controls the expression of the monocarboxylate transporter MCT4 in cultured mouse cortical astrocytes via a hypoxia-inducible factor-1alpha-mediated transcriptional regulation. *Glia* 62 477–490. 10.1002/glia.22618 24375723

[B174] Rose E. M. Koo J. C. Antflick J. E. Ahmed S. M. Angers S. Hampson D. R. (2009). Glutamate transporter coupling to Na,K-ATPase. *J. Neurosci.* 29 8143–8155. 10.1523/jneurosci.1081-09.2009 19553454 PMC6666056

[B175] Sa J. V. Kleiderman S. Brito C. Sonnewald U. Leist M. Teixeira A. P. (2017). Quantification of metabolic rearrangements during neural stem cells differentiation into astrocytes by metabolic flux analysis. *Neurochem. Res.* 42 244–253. 10.1007/s11064-016-1907-z 27068034

[B176] Sagara J. I. Miura K. Bannai S. (1993). Maintenance of neuronal glutathione by glial cells. *J. Neurochem.* 61 1672–1676. 10.1111/j.1471-4159.1993.tb09802.x 8228986

[B177] Santos C. Pai Y. J. Mahmood M. R. Leung K. Y. Savery D. Waddington S. N. (2020). Impaired folate 1-carbon metabolism causes formate-preventable hydrocephalus in glycine decarboxylase-deficient mice. *J. Clin. Invest.* 130 1446–1452. 10.1172/jci132360 31794432 PMC7269562

[B178] Sarret C. Ashkavand Z. Paules E. Dorboz I. Pediaditakis P. Sumner S. (2019). Deleterious mutations in ALDH1L2 suggest a novel cause for neuro-ichthyotic syndrome. *NPJ Genom. Med.* 4:17. 10.1038/s41525-019-0092-9 PMC6650503 31341639

[B179] Sayre N. L. Sifuentes M. Holstein D. Cheng S. Y. Zhu X. Lechleiter J. D. (2017). Stimulation of astrocyte fatty acid oxidation by thyroid hormone is protective against ischemic stroke-induced damage. *J. Cereb. Blood Flow Metab.* 37 514–527. 10.1177/0271678x16629153 26873887 PMC5381439

[B180] Schmoll D. Fuhrmann E. Gebhardt R. Hamprecht B. (1995). Significant amounts of glycogen are synthesized from 3-carbon compounds in astroglial primary cultures from mice with participation of the mitochondrial phosphoenolpyruvate carboxykinase isoenzyme. *Eur. J. Biochem.* 227 308–315. 10.1111/j.1432-1033.1995.tb20390.x 7851401

[B181] Schonfeld P. Reiser G. (2013). Why does brain metabolism not favor burning of fatty acids to provide energy? Reflections on disadvantages of the use of free fatty acids as fuel for brain. *J. Cereb. Blood Flow Metab.* 33 1493–1499. 10.1038/jcbfm.2013.128 23921897 PMC3790936

[B182] Schonfeld P. Reiser G. (2016). Brain lipotoxicity of phytanic acid and very long-chain fatty acids. harmful cellular/mitochondrial activities in Refsum disease and X-linked adrenoleukodystrophy. *Aging Dis.* 7 136–149. 10.14336/ad.2015.0823 27114847 PMC4809606

[B183] Schonfeld P. Reiser G. (2017). Brain energy metabolism spurns fatty acids as fuel due to their inherent mitotoxicity and potential capacity to unleash neurodegeneration. *Neurochem. Int.* 109 68–77. 10.1016/j.neuint.2017.03.018 28366720

[B184] Schonfeld P. Wieckowski M. R. Lebiedzinska M. Wojtczak L. (2010). Mitochondrial fatty acid oxidation and oxidative stress: lack of reverse electron transfer-associated production of reactive oxygen species. *Biochim. Biophys. Acta* 1797 929–938. 10.1016/j.bbabio.2010.01.010 20085746

[B185] Schonfeld P. Wojtczak L. (2007). Fatty acids decrease mitochondrial generation of reactive oxygen species at the reverse electron transport but increase it at the forward transport. *Biochim. Biophys. Acta* 1767 1032–1040. 10.1016/j.bbabio.2007.04.005 17588527

[B186] Schonfeld P. Wojtczak L. (2008). Fatty acids as modulators of the cellular production of reactive oxygen species. *Free Radic. Biol. Med.* 45 231–241. 10.1016/j.freeradbiomed.2008.04.029 18482593

[B187] Schonfeld P. Wojtczak L. (2016). Short- and medium-chain fatty acids in energy metabolism: the cellular perspective. *J. Lipid Res.* 57 943–954. 10.1194/jlr.r067629 27080715 PMC4878196

[B188] Schousboe A. Bak L. K. Waagepetersen H. S. (2013). Astrocytic Control of biosynthesis and turnover of the neurotransmitters glutamate and GABA. *Front. Endocrinol.* 4:102. 10.3389/fncel.2015.00102 23966981 PMC3744088

[B189] Seib T. M. Patel S. A. Bridges R. J. (2011). Regulation of the system x(C)- cystine/glutamate exchanger by intracellular glutathione levels in rat astrocyte primary cultures. *Glia* 59 1387–1401. 10.1002/glia.21176 21590811

[B190] Seifert E. L. Estey C. Xuan J. Y. Harper M. E. (2010). Electron transport chain-dependent and -independent mechanisms of mitochondrial H2O2 emission during long-chain fatty acid oxidation. *J. Biol. Chem.* 285 5748–5758. 10.1074/jbc.m109.026203 20032466 PMC2820802

[B191] Shank R. P. Bennett G. S. Freytag S. O. Campbell G. L. (1985). Pyruvate carboxylase: an astrocyte-specific enzyme implicated in the replenishment of amino acid neurotransmitter pools. *Brain Res.* 329 364–367. 10.1016/0006-8993(85)90552-9 3884090

[B192] Shanker G. Allen J. W. Mutkus L. A. Aschner M. (2001). The uptake of cysteine in cultured primary astrocytes and neurons. *Brain Res.* 902 156–163. 10.1016/s0006-8993(01)02342-3 11384608

[B193] Shih A. Y. Johnson D. A. Wong G. Kraft A. D. Jiang L. Erb H. (2003). Coordinate regulation of glutathione biosynthesis and release by Nrf2-expressing glia potently protects neurons from oxidative stress. *J. Neurosci.* 23 3394–3406. 10.1523/jneurosci.23-08-03394.2003 12716947 PMC6742304

[B194] Sibson N. R. Dhankhar A. Mason G. F. Rothman D. L. Behar K. L. Shulman R. G. (1998). Stoichiometric coupling of brain glucose metabolism and glutamatergic neuronal activity. *Proc. Natl. Acad. Sci. U.S.A.* 95 316–321. 10.1073/pnas.95.1.316 9419373 PMC18211

[B195] Sierra A. Y. Gratacos E. Carrasco P. Clotet J. Urena J. Serra D. (2008). CPT1c is localized in endoplasmic reticulum of neurons and has carnitine palmitoyltransferase activity. *J. Biol. Chem.* 283 6878–6885. 10.1074/jbc.m707965200 18192268

[B196] Smith D. Pernet A. Hallett W. A. Bingham E. Marsden P. K. Amiel S. A. (2003). Lactate: a preferred fuel for human brain metabolism in vivo. *J. Cereb. Blood Flow Metab.* 23 658–664. 10.1097/01.wcb.0000063991.19746.11 12796713

[B197] Song H. Stevens C. F. Gage F. H. (2002). Astroglia induce neurogenesis from adult neural stem cells. *Nature* 417 39–44. 10.1038/417039a 11986659

[B198] Sonnewald U. Westergaard N. Petersen S. B. Unsgard G. Schousboe A. (1993). Metabolism of [U-13C]glutamate in astrocytes studied by 13C NMR spectroscopy: incorporation of more label into lactate than into glutamine demonstrates the importance of the tricarboxylic acid cycle. *J. Neurochem.* 61 1179–1182. 10.1111/j.1471-4159.1993.tb03641.x 8103082

[B199] Stark R. Kibbey R. G. (2014). The mitochondrial isoform of phosphoenolpyruvate carboxykinase (PEPCK-M) and glucose homeostasis: has it been overlooked? *Biochim. Biophys. Acta* 1840 1313–1330. 10.1016/j.bbagen.2013.10.033 24177027 PMC3943549

[B200] Stark R. Pasquel F. Turcu A. Pongratz R. L. Roden M. Cline G. W. (2009). Phosphoenolpyruvate cycling via mitochondrial phosphoenolpyruvate carboxykinase links anaplerosis and mitochondrial GTP with insulin secretion. *J. Biol. Chem.* 284 26578–26590. 10.1074/jbc.m109.011775 19635791 PMC2785346

[B201] Stephen T. L. Higgs N. F. Sheehan D. F. Al Awabdh S. Lopez-Domenech G. Arancibia-Carcamo I. L. (2015). Miro1 regulates activity-driven positioning of mitochondria within astrocytic processes apposed to synapses to regulate intracellular calcium signaling. *J. Neurosci.* 35 15996–16011. 10.1523/jneurosci.2068-15.2015 26631479 PMC4666922

[B202] Sugiura A. Mattie S. Prudent J. Mcbride H. M. (2017). Newly born peroxisomes are a hybrid of mitochondrial and ER-derived pre-peroxisomes. *Nature* 542 251–254. 10.1038/nature21375 28146471

[B203] Sun X. Shih A. Y. Johannssen H. C. Erb H. Li P. Murphy T. H. (2006). Two-photon imaging of glutathione levels in intact brain indicates enhanced redox buffering in developing neurons and cells at the cerebrospinal fluid and blood-brain interface. *J. Biol. Chem.* 281 17420–17431. 10.1074/jbc.m601567200 16624809

[B204] Supplie L. M. Duking T. Campbell G. Diaz F. Moraes C. T. Gotz M. (2017). Respiration-deficient astrocytes survive as glycolytic cells in vivo. *J. Neurosci.* 37 4231–4242. 10.1523/jneurosci.0756-16.2017 28314814 PMC6596567

[B205] Takahashi S. Iizumi T. Mashima K. Abe T. Suzuki N. (2014). Roles and regulation of ketogenesis in cultured astroglia and neurons under hypoxia and hypoglycemia. *ASN Neuro* 6:1759091414550997. 10.1177/1759091414550997 PMC4187005 25290061

[B206] Thevenet J. De Marchi U. Domingo J. S. Christinat N. Bultot L. Lefebvre G. (2016). Medium-chain fatty acids inhibit mitochondrial metabolism in astrocytes promoting astrocyte-neuron lactate and ketone body shuttle systems. *FASEB J.* 30 1913–1926. 10.1096/fj.201500182 26839375

[B207] Troffer-Charlier N. Doerflinger N. Metzger E. Fouquet F. Mandel J. L. Aubourg P. (1998). Mirror expression of adrenoleukodystrophy and adrenoleukodystrophy related genes in mouse tissues and human cell lines. *Eur. J. Cell Biol.* 75 254–264. 10.1016/s0171-9335(98)80121-0 9587057

[B208] Trompier D. Vejux A. Zarrouk A. Gondcaille C. Geillon F. Nury T. (2014). Brain peroxisomes. *Biochimie* 98 102–110. 10.1016/j.biochi.2013.09.009 24060512

[B209] Valdebenito R. Ruminot I. Garrido-Gerter P. Fernandez-Moncada I. Forero-Quintero L. Alegria K. (2016). Targeting of astrocytic glucose metabolism by beta-hydroxybutyrate. *J. Cereb. Blood Flow Metab.* 36 1813–1822. 10.1177/0271678x15613955 26661221 PMC5076786

[B210] van Hall G. Stromstad M. Rasmussen P. Jans O. Zaar M. Gam C. (2009). Blood lactate is an important energy source for the human brain. *J. Cereb. Blood Flow Metab.* 29 1121–1129. 10.1038/jcbfm.2009.35 19337275

[B211] Vangeison G. Carr D. Federoff H. J. Rempe D. A. (2008). The good, the bad, and the cell type-specific roles of hypoxia inducible factor-1 alpha in neurons and astrocytes. *J. Neurosci.* 28 1988–1993. 10.1523/jneurosci.5323-07.2008 18287515 PMC6671445

[B212] Vasquez O. L. Almeida A. Bolanos J. P. (2001). Depletion of glutathione up-regulates mitochondrial complex I expression in glial cells. *J. Neurochem.* 76 1593–1596. 10.1046/j.1471-4159.2001.00223.x 11238744

[B213] Vaughn A. E. Deshmukh M. (2008). Glucose metabolism inhibits apoptosis in neurons and cancer cells by redox inactivation of cytochrome c. *Nat. Cell Biol.* 10 1477–1483. 10.1038/ncb1807 19029908 PMC2626347

[B214] Verkhratsky A. Matteoli M. Parpura V. Mothet J. P. Zorec R. (2016). Astrocytes as secretory cells of the central nervous system: idiosyncrasies of vesicular secretion. *EMBO J.* 35 239–257. 10.15252/embj.201592705 26758544 PMC4741299

[B215] Vicente-Gutierrez C. Bonora N. Bobo-Jimenez V. Jimenez-Blasco D. Lopez-Fabuel I. Fernandez E. (2019). Astrocytic mitochondrial ROS modulate brain metabolism and mouse behaviour. *Nat. Metab.* 1 201–211. 10.1038/s42255-018-0031-6 32694785

[B216] Vitvitsky V. Thomas M. Ghorpade A. Gendelman H. E. Banerjee R. (2006). A functional transsulfuration pathway in the brain links to glutathione homeostasis. *J. Biol. Chem.* 281 35785–35793. 10.1074/jbc.m602799200 17005561

[B217] Vogel R. Hamprecht B. Wiesinger H. (1998). Malic enzyme isoforms in astrocytes: comparative study on activities in rat brain tissue and astroglia-rich primary cultures. *Neurosci. Lett.* 247 123–126. 10.1016/s0304-3940(98)00290-0 9655608

[B218] Vogel R. Wiesinger H. Hamprecht B. Dringen R. (1999). The regeneration of reduced glutathione in rat forebrain mitochondria identifies metabolic pathways providing the NADPH required. *Neurosci. Lett.* 275 97–100. 10.1016/s0304-3940(99)00748-x 10568508

[B219] Voloboueva L. A. Suh S. W. Swanson R. A. Giffard R. G. (2007). Inhibition of mitochondrial function in astrocytes: implications for neuroprotection. *J. Neurochem.* 102 1383–1394. 10.1111/j.1471-4159.2007.04634.x 17488276 PMC3175820

[B220] von Bartheld C. S. Bahney J. Herculano-Houzel S. (2016). The search for true numbers of neurons and glial cells in the human brain: a review of 150 years of cell counting. *J. Comp. Neurol.* 524 3865–3895. 27187682 10.1002/cne.24040 PMC5063692

[B221] von Bernhardi R. Eugenin-Von Bernhardi J. Flores B. Eugenin Leon J. (2016). Glial cells and integrity of the nervous system. *Adv. Exp. Med. Biol.* 949 1–24. 10.1007/978-3-319-40764-7_1 27714682

[B222] Wang W. Fang H. Groom L. Cheng A. Zhang W. Liu J. (2008). Superoxide flashes in single mitochondria. *Cell* 134 279–290. 18662543 10.1016/j.cell.2008.06.017 PMC2547996

[B223] Wang X. F. Cynader M. S. (2000). Astrocytes provide cysteine to neurons by releasing glutathione. *J. Neurochem.* 74 1434–1442. 10.1046/j.1471-4159.2000.0741434.x 10737599

[B224] Weber B. Barros L. F. (2015). The astrocyte: powerhouse and recycling center. *Cold Spring Harb. Perspect. Biol.* 7:a020396. 10.1101/cshperspect.a020396 25680832 PMC4665076

[B225] Weightman Potter P. G. Vlachaki Walker J. M. Robb J. L. Chilton J. K. Williamson R. Randall A. D. (2019). Basal fatty acid oxidation increases after recurrent low glucose in human primary astrocytes. *Diabetologia* 62 187–198. 10.1007/s00125-018-4744-6 30293112 PMC6290858

[B226] Wolfgang M. J. Kurama T. Dai Y. Suwa A. Asaumi M. Matsumoto S. (2006). The brain-specific carnitine palmitoyltransferase-1c regulates energy homeostasis. *Proc. Natl. Acad. Sci. U.S.A.* 103 7282–7287. 10.1073/pnas.0602205103 16651524 PMC1564279

[B227] Wong-Riley M. T. (1989). Cytochrome oxidase: an endogenous metabolic marker for neuronal activity. *Trends Neurosci.* 12 94–101. 10.1016/0166-2236(89)90165-3 2469224

[B228] Wu D. Pardridge W. M. (1999). Blood-brain barrier transport of reduced folic acid. *Pharm. Res.* 16 415–419. 10213373 10.1023/a:1018829920158

[B229] Yang M. Vousden K. H. (2016). Serine and one-carbon metabolism in cancer. *Nat. Rev. Cancer* 16 650–662. 10.1038/nrc.2016.81 27634448

[B230] Yang S. Y. He X. Y. Schulz H. (1987). Fatty acid oxidation in rat brain is limited by the low activity of 3-ketoacyl-coenzyme A thiolase. *J. Biol. Chem.* 262 13027–13032. 3654601

[B231] Yang Y. Gozen O. Watkins A. Lorenzini I. Lepore A. Gao Y. (2009). Presynaptic regulation of astroglial excitatory neurotransmitter transporter GLT1. *Neuron* 61 880–894. 10.1016/j.neuron.2009.02.010 19323997 PMC2743171

[B232] Yao C. H. Liu G. Y. Wang R. Moon S. H. Gross R. W. Patti G. J. (2018). Identifying off-target effects of etomoxir reveals that carnitine palmitoyltransferase I is essential for cancer cell proliferation independent of beta-oxidation. *PLoS Biol.* 16:e2003782. 10.1371/journal.pbio.2003782 29596410 PMC5892939

[B233] Yip J. Geng X. Shen J. Ding Y. (2016). Cerebral gluconeogenesis and diseases. *Front. Pharmacol.* 7:521. 10.3389/fncel.2015.00521 28101056 PMC5209353

[B234] Yu A. C. Drejer J. Hertz L. Schousboe A. (1983). Pyruvate carboxylase activity in primary cultures of astrocytes and neurons. *J. Neurochem.* 41 1484–1487. 10.1111/j.1471-4159.1983.tb00849.x 6619879

[B235] Zielke H. R. Zielke C. L. Baab P. J. (2009). Direct measurement of oxidative metabolism in the living brain by microdialysis: a review. *J. Neurochem.* 109(Suppl. 1) 24–29. 10.1111/j.1471-4159.2009.05941.x 19393005 PMC2792919

